# H3K27ac Is Essential for Human Naive Pluripotency Modulated by m6A‐Driven EP300 Expression

**DOI:** 10.1002/advs.202507549

**Published:** 2025-08-11

**Authors:** Chenrui An, Ke Zhong, Xiangjin Kang, Yingying Zhang, Yudan Shang, Hao Liu, Yushan Ji, Jinbei Zeng, Jiajin Lin, Long Jin, Zelong Lin, Yang Yu, Xuetao Shi, Yong Fan

**Affiliations:** ^1^ Department of Obstetrics and Gynecology Guangdong Provincial Key Laboratory of Major Obstetric Diseases Guangdong Provincial Clinical Research Center for Obstetrics and Gynecology Guangdong‐Hong Kong‐Macao Greater Bay Area Higher Education Joint Laboratory of Maternal‐Fetal Medicine The Third Affiliated Hospital Guangzhou Medical University Guangzhou 510150 China; ^2^ Beijing Key Laboratory of Reproductive Endocrinology and Assisted Reproductive Technology and Key Laboratory of Assisted Reproduction Ministry of Education Center of Reproductive Medicine Department of Obstetrics and Gynecology Stem Cell Research Center Peking University Third Hospital Beijing 100191 China; ^3^ Stem Cell Research Center Peking University Third Hospital Beijing 100191 China; ^4^ National Engineering Research Centre for Tissue Restoration and Reconstruction and Key Laboratory of Biomedical Engineering of Guangdong Province South China University of Technology Guangzhou 510640 China; ^5^ Key Laboratory of Biomedical Engineering of Guangdong Province South China University of Technology Guangzhou 510006 China; ^6^ School of Materials Science and Engineering South China University of Technology Guangzhou 510640 China

**Keywords:** EP300, H3K27ac, histone acetylation, human naive pluripotency, m6A

## Abstract

Human pluripotent stem cells (hPSCs) can transition between primed and naive states, each defined by unique epigenetic features crucial for early human development. However, the precise regulatory mechanisms of these transitions, particularly involving the roles of H3K27ac and m6A modifications, remain unclear. Here, it is revealed that H3K27ac is essential for establishment of human naive pluripotency. This elevation is critical for maintaining the naive state, as inhibiting H3K27ac disrupts naive pluripotency, while enhancing it promotes the naive state. Furthermore, it is discovered that m6A, via the enzyme METTL3, indirectly controls H3K27ac by regulating EP300 expression, an enzyme involved in H3K27ac deposition. *METTL3* overexpression suppresses EP300 protein, thereby reducing H3K27ac modification and impeding naive pluripotency. These findings uncover a novel interplay between H3K27ac and m6A, advancing the understanding of stem cell regulation. This work provides new insights into the epigenetic control of hPSCs, with potential implications for developmental biology and regenerative medicine.

## Graphical abstract

An et al. reveal H3K27ac elevation is essential for establishing and maintaining human naive pluripotency; its inhibition disrupts this state while enhancement promotes it. METTL3‐mediated m6A modification indirectly controls H3K27ac by suppressing EP300 expression. Reduced EP300 lowers H3K27ac, impeding naive pluripotency. This uncovers a critical METTL3‐EP300‐H3K27ac interplay regulating human naive pluripotency.

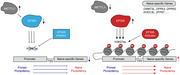

## 1 Introduction

Pluripotent stem cells can differentiate into cells of all three germ layers, offering significant potential for developmental biology and regenerative medicine.^[^ ^1^ ^]^ Two distinct pluripotency states, naive and primed, have been defined in the mouse embryo. Mouse embryonic stem cells (mESCs), derived from the preimplantation epiblast, represent the naive pluripotent state and transition into primed mouse epiblast stem cells (mEpiSCs) after implantation.^[^ ^2^ ^]^ However, human embryonic stem cells (hESCs) were initially isolated in a primed state, resembling post‐implantation epiblast cells.^[^ ^2^, ^3^ ^]^ In the past decade, various protocols have established naive hPSCs using combinations of inhibitors and growth factors,^[^ ^4^ ^]^ producing cells resembling early preimplantation epiblast with dome‐shaped colonies. Human extended pluripotent stem cells (hEPSCs), with broader differentiation potential, have also been established, expanding the understanding of pluripotency.^[^ ^5^ ^]^ Key differences between naive and primed states include morphology, culture conditions, transcriptional profiles, and, notably, epigenetic landscapes, such as X chromosome reactivation, global genomic hypomethylation, and histone modification remodeling.^[^ ^1^, ^2^, ^3^, ^6^ ^]^ Despite advancements, the precise mechanisms controlling the transitions between these pluripotent states remain largely unknown.

Protein acetylation, an essential post‐translational modification, regulates various cellular functions like cell survival, proliferation, metabolism, and gene expression.^[^ ^7^ ^]^ Acetylation adds acetyl groups to lysine residues on histone and non‐histone proteins, neutralizing the positive charge, which results in relaxed chromatin that promotes transcription.^[^ ^8^ ^]^ In PSCs, acetylation is essential for balancing self‐renewal and differentiation.^[^ ^9^ ^]^ In mESCs, protein acetylation supports the naive state by keeping chromatin open and enabling efficient transcription of pluripotency‐associated genes.^[^ ^10^ ^]^ Research on hPSCs indicates that acetylation affects both naive and primed states, modulating chromatin accessibility and transcriptional networks essential for pluripotency.^[^ ^11^ ^]^ However, specific studies on protein acetylation's role in human naive and primed states remain limited, underscoring the need for further research into its regulation of dynamic transitions between these states.

Histone acetylation, particularly at lysine 27 on histone H3 (H3K27ac), is essential for gene regulation during early embryogenesis and maintaining pluripotency.^[^ ^12^ ^]^ H3K27ac, an epigenetic mark at active enhancer regions, promotes transcription by enhancing chromatin accessibility.^[^ ^13^ ^]^ EP300 acetyltransferase mediates H3K27 acetylation, crucial for sustaining pluripotency transcriptional programs.^[^ ^14^ ^]^ Comparisons between naive and primed pluripotent stem cells reveal that naive cells have higher H3K27ac levels, particularly at pluripotency‐regulating loci.^[^ ^13^, ^15^ ^]^ In naive hPSCs, H3K27ac marks key regulatory regions that maintain the naive state, whereas in primed cells, H3K27ac levels are lower. Despite progress, the mechanisms by which H3K27ac regulate transitions between naive and primed states in hPSCs are still underexplored.

m6A is the most common internal modification on eukaryotic mRNAs and is crucial for regulating gene expression, mRNA stability, splicing, and translation.^[^ ^16^ ^]^ m6A has emerged as a key regulator of cell fate decisions in early embryonic development, modulating mRNA decay to ensure proper gene expression.^[^ ^17^ ^]^ In mouse and human PSCs, m6A regulates the balance between self‐renewal and differentiation,^[^ ^18^ ^]^ with specific m6A patterns distinguishing naive from primed states.^[^ ^17^, ^19^ ^]^ The METTL3 methyltransferase complex adds m6A marks,^[^ ^20^ ^]^ with activity crucial for maintaining the naive state by stabilizing and translating pluripotency‐related transcripts. Despite recent progress, the precise molecular mechanisms by which m6A regulate transitions between naive and primed pluripotency, especially in hPSCs, remain unclear.

Here, we demonstrate that EP300‐mediated H3K27ac and *METTL3*‐driven m6A modifications are essential for maintaining human naive pluripotency, revealing novel epigenetic interactions between histone acetylation and RNA methylation. Our findings indicate that naive hPSCs exhibit higher H3K27ac levels than primed cells, with active acetylation at key pluripotency regulatory sites. Notably, we found that m6A, through *METTL3*, regulates EP300 expression and indirectly influences H3K27ac deposition, indicating a new layer of epigenetic regulation in pluripotency transitions. This regulatory axis stabilizes key pluripotency genes and fine‐tunes chromatin accessibility, setting the naive state apart from the primed state. These insights deepen our understanding of the molecular mechanisms driving naive pluripotency and broaden the potential for therapeutic applications in regenerative medicine.

## 2 Results

### 2.1 Diversity in Post‐translational Acetylation among Human Primed, Naive, and Extended Pluripotent Stem Cells

We generated naive hiPSCs using the HENSM resetting system,^[^ ^4l^ ^]^ and hEPSCs using the LCDM system^[^ ^5b^ ^]^ (Figure S1A, Supporting Information). Dome‐shaped naive hiPSCs and hEPSCs exhibited high alkaline phosphatase activity (Figure S1B, Supporting Information) and expression levels of OCT4 (Figure S1C, Supporting Information). Naïve pluripotent marker genes significantly increased in the naive hiPSCs, compared to primed hiPSCs and hEPSCs (Figure S1C, Supporting Information).

**Figure 1:**
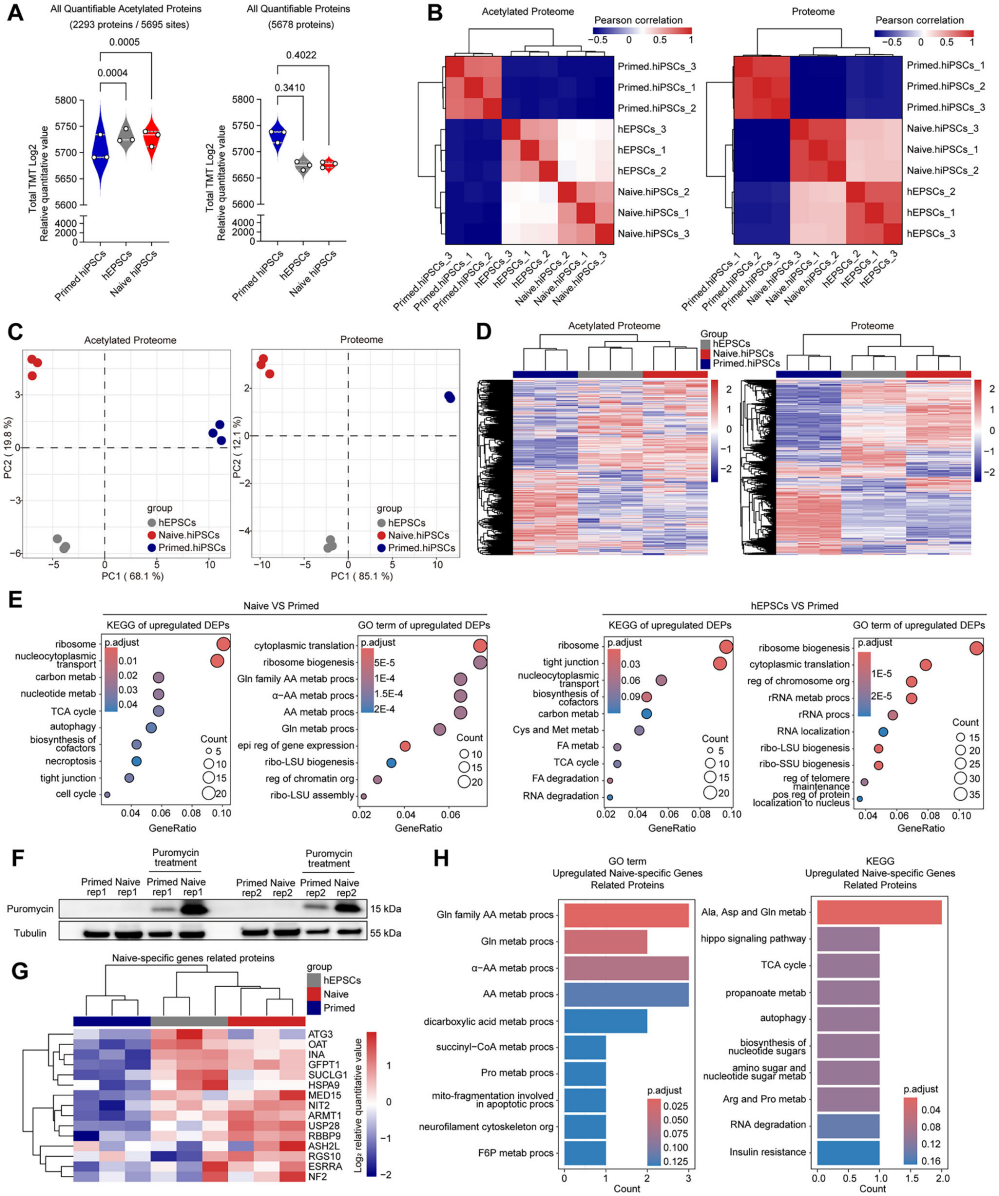
Post‐translational acetylation in human primed, naive, and extended pluripotent stem cells. A) Quantitative analysis of acetylated (left panel) and total proteins (right panel) in primed and naive hiPSCs, and hEPSCs. *P*‐values showing the significance between naive and primed hiPSCs, or between hEPSCs and primed hiPSCs. B) Correlation coefficient analysis of the acetylated (left panel) and total proteomes (right panel) showing distinct profiles between primed and naive hiPSCs, and hEPSCs. C) PCA of the acetylated (left panel) and total proteomes (right panel) in the three types of hPSCs. D) Hierarchical clustering analysis showing the separated acetylated (left panel) and total protein (right panel) profiles among primed and naive hiPSCs, and hEPSCs. E) KEGG pathway and GO term analysis of differentially expressed acetylated proteins in naive hiPSCs and hEPSCs compared to primed hiPSCs. F) Western blot analysis showing the puromycin levels between naive and primed hiPSCs. G) Heatmap showing the upregulated acetylated proteins encoded by naive‐specific genes. H) GO term and KEGG pathway analysis of the upregulated naive‐specific genes.

To explore differential protein acetylation among pluripotent stem cells, we trypsinized total proteins from primed and naive hiPSCs and hEPSCs, labeled them with Tandem Mass Tags (TMT), fractionated them by high‐performance liquid chromatography, enriched acetylation‐modified peptides, and conducted high‐performance liquid chromatography‐mass spectrometry (HPLC‐MS) analysis. We performed a quantitative proteomic analysis on acetylated and total proteins in different hiPSCs. In total, 2293 quantifiable acetylated proteins and 5678 proteins were detected (Figure S1D, Supporting Information). The detected acetylated sites widely distributed in subcellular structures, mainly in the cytoplasm and nucleus (Figure S1E, Supporting Information). Naive hiPSCs and hEPSCs exhibited higher levels of acetylated proteins than primed hiPSCs (**Figure** **1**A), suggesting more active protein acetylation in naive and extended states. No significant differences were observed in total protein content among these three types of pluripotent stem cells (Figure 1A). Correlation coefficient and principal component analyses revealed significant differences in the acetylated and total proteomes among the three pluripotent stem cells, with no significant variation among biological replicates within each sample (Figure 1B,C). Hierarchical clustering of acetylated and total protein expression distinguished these three types of pluripotent stem cells, with the largest difference observed between naive and primed hiPSCs (Figure 1D). In both acetylated and total proteomes, hEPSCs were more similar to naive hiPSCs (Figure 1D). These results indicate significant differential acetylation modifications among hPSCs.

To further explore differential acetylation modifications among these hPSCs, we analyzed differentially expressed acetylated proteins between samples (Figure S1F, Supporting Information). Functional annotation using Gene Ontology (GO) Term and Kyoto Encyclopedia of Genes and Genomes (KEGG) showed that compared to primed hiPSCs, both naive hiPSCs and hEPSCs significantly upregulated pathways related to ribosome synthesis (Figure 1E). These suggest that naive hiPSCs and hEPSCs may enhance protein translation through increased acetylation. Clusters of Orthologous Groups of proteins (COGs) functional analysis showed similar results; naive hiPSCs and hEPSCs significantly upregulated acetylation of post‐translational modification‐related proteins compared to primed hiPSCs (Figure S1G, Supporting Information). To test this, we performed the puromycin incorporating on primed and naive hiPSCs. After 30 min of treatment, naive hiPSCs exhibited higher levels of puromycin than primed hiPSCs (Figure 1F and Figure S1J, Supporting Information), demonstrating the stronger protein translation activity of naive hiPSCs.

To examine the impact of acetylation modification on human naive pluripotency, we summarized core genes specifically upregulated in naive or primed states, respectively, beyond the well‐known pluripotent genes. We analyzed RNA‐seq data from ten previous studies using various methods to establish human naive pluripotency^[^ ^4^, ^6^, ^21^ ^]^ (Figure S1H, Supporting Information). Integration of differentially expressed genes between naive and primed hPSCs across these studies, we identified 228 genes specifically upregulated in naive hPSCs, and 296 in primed hPSCs (Figure S1I, Supporting Information). By screening using these naive‐specific upregulated genes, we found 15 proteins encoded by these genes in the detected acetylated proteins. All these 15 proteins were highly acetylated in naive hiPSCs, compared to primed hiPSCs (Figure 1G). These proteins are associated with the tricarboxylic acid cycle and autophagy (Figure 1H), which are significantly enhanced in naive hPSCs.^[^ ^22^ ^]^ When treated with Garcinol, a polyisoprenylated benzophenone inhibiting histone acetyltransferases and p300/CPB‐associated factor, the expression level of ATG3 protein was dramatically downregulated (Figure S1K, Supporting Information), indicating the pivot role of the acetylation modification on this protein.

### 2.2 Differential Quantification of Histone H3 Acetylation at Lysine 27 between Human Naive and Primed Pluripotent Stem Cells

**Figure 2:**
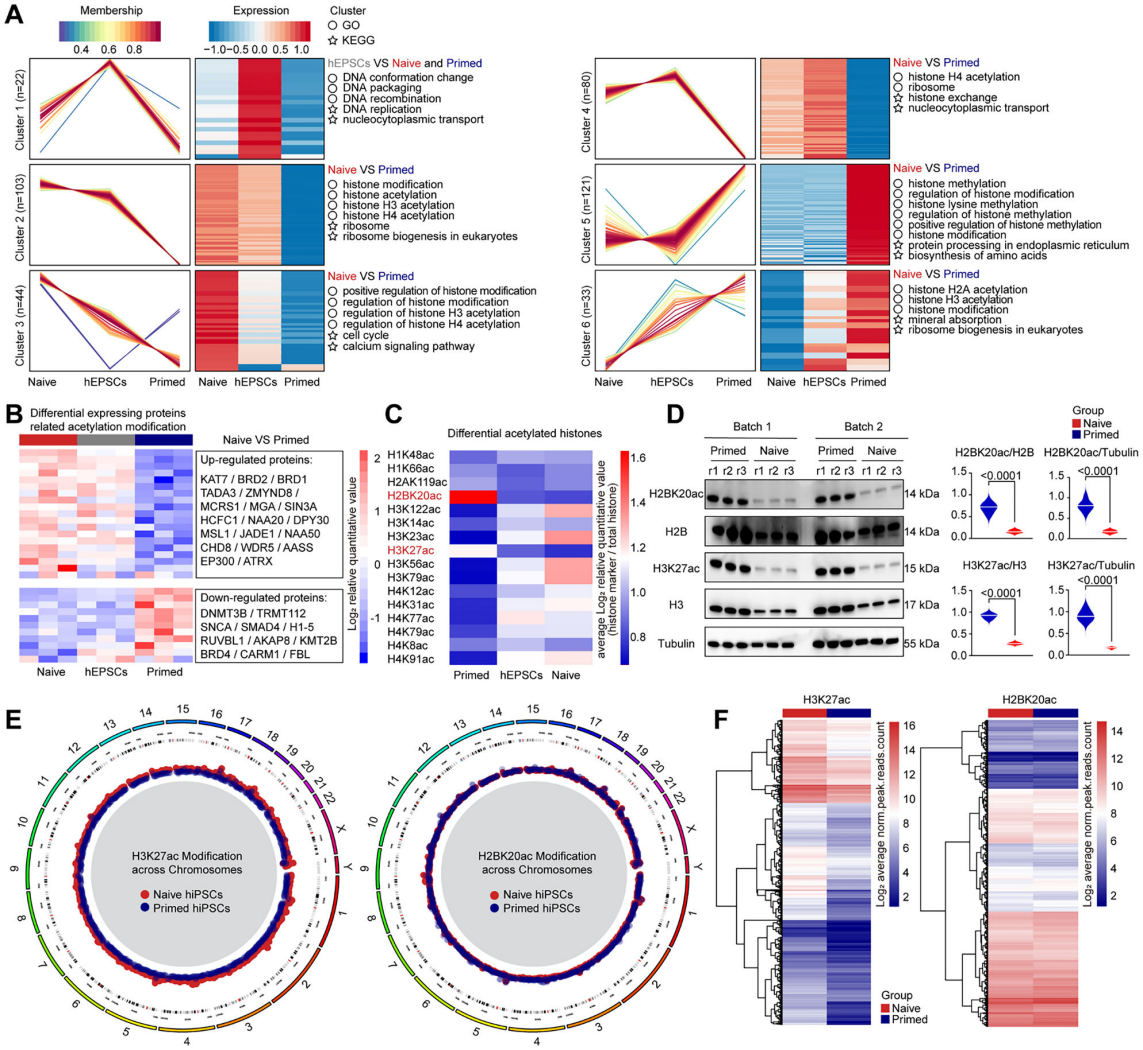
Differential histone acetylation between naive and primed hiPSCs. A) Line plots and heatmaps showing acetylated proteins in primed and naive hiPSCs, and hEPSCs, categorized into six clusters. GO term (circle) and KEGG pathway (star) analysis of the clustered proteins are listing on the right of heatmaps. B) Differential expression patterns of histone acetylation writer and eraser proteins among three types of hPSCs. C) Heatmap displaying all the detected acetylated histones of three types of hPSCs. D) Western blot validation of H2BK20ac and H3K27ac levels in naive and primed hiPSCs (left panel) and quantified with H2B, H3, and Tubulin. E) H3K27ac peaks (left panel) and H2BK20ac peaks (right panel) across the chromosomes. Each red (naive hiPSCs) or blue (primed hiPSCs) dot represent a peak. F) Heatmaps showing all the detected H3K27ac (left panel) and H2BK20ac (right panel) peaks between naive and primed hiPSCs. Each line represents a peak. The levels of peaks were calculated using Log_2_ (average of normalized peak counts).

We clustered the acetylated protein profiles of the three types of pluripotent stem cell based on the expression patterns and categorized into six groups (**Figure** **2**A). GO Term and KEGG functional annotations showed that differential expressed acetylated proteins between naive and primed hiPSCs were mainly enriched in histone acetylation (Figure 2A and Figure S2A, Supporting Information). Only the differentially acetylated proteins in Cluster 1 were significantly upregulated in hEPSCs, with functions primarily related to DNA replication, recombination, and packaging (Figure 2A). By assessing the expression levels of histone acetylation related proteins, we found that naive hiPSCs were significantly upregulated compared to primed hiPSCs (Figure 2B), especially the proteins encoded by the histone acetylation writer genes (Figure S2B, Supporting Information), whereas no obvious differences in the histone acetylation eraser gene coding proteins (Figure S2C, Supporting Information).

Comprehensive analysis of all detectable acetylated histones revealed multiple differential modifications between naive and primed hiPSCs (Figure 2C). The expression levels of most of the acetylated histones were higher in naive hiPSCs, compared to primed hiPSCs (Figure 2C), which may be due to the more powerful protein translation activity and post‐translational modification of naive hiPSCs. Among these, only acetylated H2B at lysine 20 (H2BK20ac) and acetylated H3 at lysine 27 (H3K27ac) exhibited higher levels in primed hiPSCs than naive hiPSCs (Figure 2C,D; Figure S2D,E, Supporting Information). We wonder whether these two histone acetylation modifications have impacts on the chromatin of naive hiPSCs.

To confirm this, we performed H2BK20ac and H3K27ac CUT&Tag sequencing on primed and naive hiPSCs. No significant differences of H2BK20ac modification were observed between naive and primed hiPSCs (Figure S2F, Supporting Information). The H2BK20ac modification across chromosomes in naive hiPSCs were highly similar to that of primed hiPSCs (Figure 2E,F), only sporadic sites showed differential modification levels (Figure S2G, Supporting Information). Surprisingly, we found genome‐wide more abundance H3K27ac modification in naive hiPSCs rather than primed hiPSCs (Figure 2E,F and Figure S2F, Supporting Information). Most of the differential H3K27ac peaks were upregulated in naive hiPSCs (Figure S2G, Supporting Information). These results seem to be somewhat contradictory to the previous finding that primed hiPSCs have more acetylated histone 3 at position 27 of lysine (Figure 2C,D), so the potential function of H3K27ac modification in naive and primed hiPSCs needs further investigation.

### 2.3 Naive hPSCs Show More Abundant H3K27ac Modification Compared to Primed hPSCs

**Figure 3:**
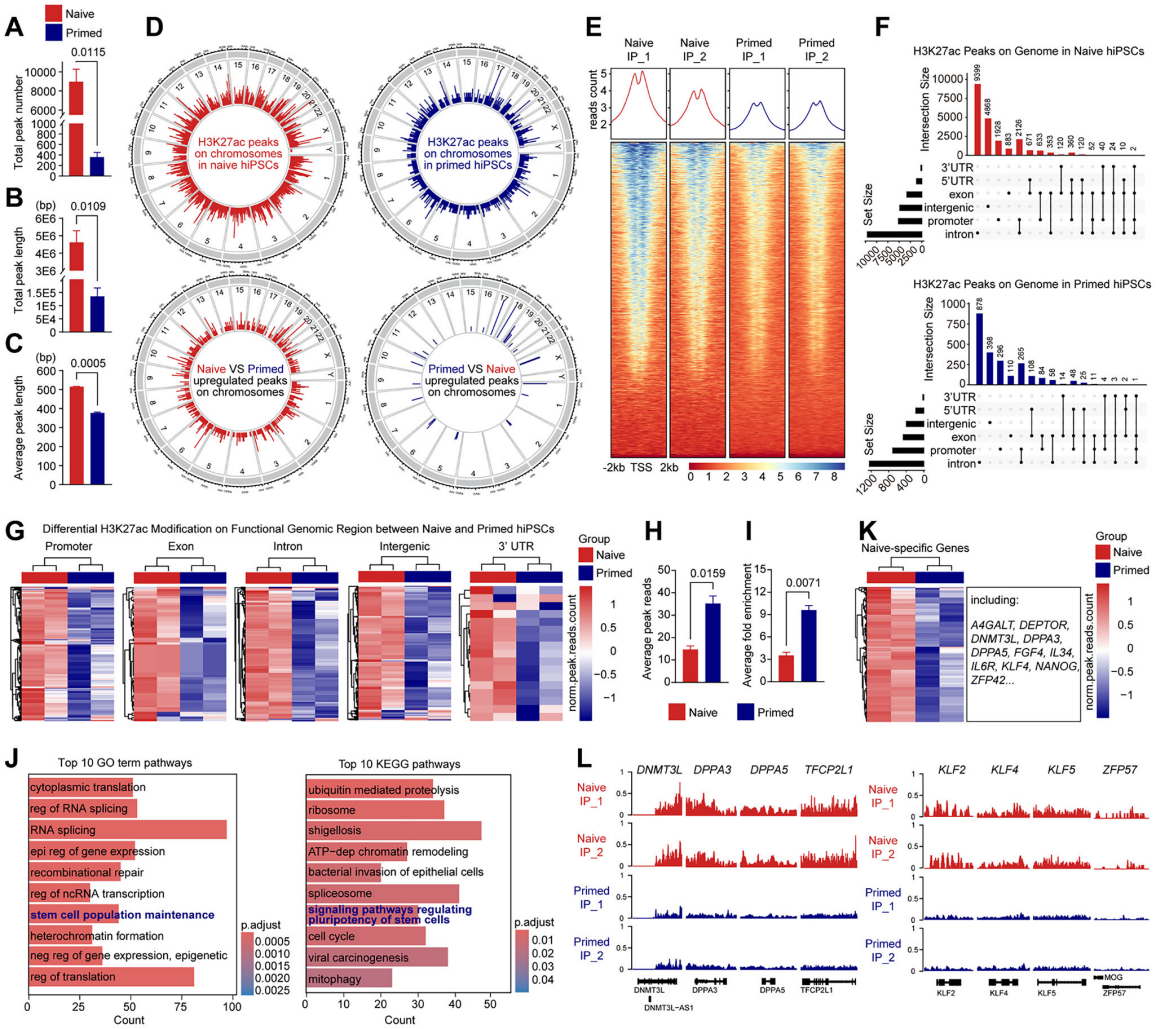
Genome‐wide H3K27ac modification in naive and primed hiPSCs. A) Histogram showing the total H3K27ac peak numbers in naive (red) and primed (blue) hiPSCs. B) Histogram displaying the total H3K27ac peak length in naive and primed hiPSCs. C) Histogram showing the average H3K27ac peak length between samples. Average peak length = Total peak length / Total peak number. D) All detected (upper panel) and differential (lower panel) H3K27ac peaks across chromosomes in naive and primed hiPSCs. Each red (naive) or blue (primed) vertical line represents a peak. E) Differential H3K27ac peaks at TSS between naive and primed hiPSCs. F,G) Distribution and modification of differentially enriched H3K27ac peaks across introns, exons, intergenic regions, promoters, and UTRs in naive and primed hiPSCs. H) Histogram showing the average H3K27ac peak reads between naive and primed hiPSCs. Average peak reads = Total peak reads / Total peak number. I) Histogram showing the average H3K27ac fold enrichment in naive and primed hiPSCs, normalized to Input DNA. Fold enrichment = (IP reads in peak / total IP reads) / (Input reads in peak / total Input reads). J) GO term and KEGG pathway analysis of genes within upregulated H3K27ac peaks in naive hiPSCs. K,L) Heatmap and IGV visualization showing the differential H3K27ac peaks in naive‐specific genes.

To further investigate differential H3K27ac modification between naive and primed hiPSCs, we conducted ChIP‐seq experiments. Consisting with the analysis of CUT&Tag sequencing results, H3K27ac peak number (**Figure** **3**A) and peak length (Figure 3B,C and Figure S3A, Supporting Information) in naive hiPSCs were significantly higher than in primed hiPSCs, indicating that naive hiPSCs exhibit more widespread H3K27ac modification than primed hiPSCs. To confirm this finding, we examined H3K27ac marks across each chromosome and found that naive hiPSCs had more H3K27ac peaks than primed hiPSCs (Figure 3D). Differential peak enrichment analysis of H3K27ac at transcription start sites (TSS) showed consistent results (Figure 3E and Figure S3B, Supporting Information). The differentially modified H3K27ac peaks were widely enriched in introns, promoters, intergenic regions, exons, and 5′ or 3′ UTRs (Figure 3F,G and Figure S3C, Supporting Information). Given that primed hiPSCs have more acetylated H3 histones at lysine 27 than naive hiPSCs (Figure 2C,D), we further calculated the average H3K27ac peak reads and fold enrichment of IP over Input in these cells. In comparison, primed hiPSCs exhibited higher H3K27ac peak intensity (average peak reads, Figure 3H) and density (average fold enrichment, Figure 3I) than naive hiPSCs. These results suggest that naive hiPSCs have a greater breadth of H3K27ac modified regions, while primed hiPSCs have a smaller overall distribution but a stronger and denser modification at limited sites.

Next, we examined whether this extensive H3K27ac modification in naive hiPSCs contributes to the maintenance of pluripotency. GO term and KEGG functional annotations on genes within the H3K27ac differential enrichment peaks, we found that genes within upregulated peaks in naive hiPSCs were associated with pluripotency maintenance (Figure 3J), while genes within upregulated peaks in primed hiPSCs were linked to lineage differentiation (Figure S3D, Supporting Information).

H3K27ac modification on naive‐specific genes was significantly stronger in naive hiPSCs compared to primed hiPSCs (Figure 3K,L). In contrast, primed pluripotent genes showed no differential H3K27ac modification between the two pluripotent states (Figure S3E, Supporting Information). These results suggest that H3K27ac may play a role in establishing human naive pluripotency as an epigenetic modification mark.

### 2.4 EP300‐Mediated H3K27ac Is Indispensable for Establishing Human Naive Pluripotency

To investigate the regulatory role of H3K27ac in human naive pluripotency, we used two small molecule compounds to modulate H3K27ac through activation or inhibition. H3K27ac is primarily regulated by EP300, with A‐485 as a significant inhibitor,^[^ ^23^ ^]^ while CTB acts as an activator of EP300.^[^ ^24^ ^]^ Primed hiPSCs were treated with varying concentrations of A‐485 or CTB. Western blot results showed that 1.5 × 10^−6^ m A‐485 treatment could reduce H3K27ac levels, while 2 × 10^−6^ m CTB increased the level (Figure S4A, Supporting Information). The primed hiPSCs under A‐485 or CTB treatment retained the flat colony morphology (Figure S4B, Supporting Information), alkaline phosphatase activity (Figure S4C, Supporting Information), and comparable expression levels of pluripotent genes to primed hiPSCs without treatment (Figure S4D, Supporting Information).

**Figure 4:**
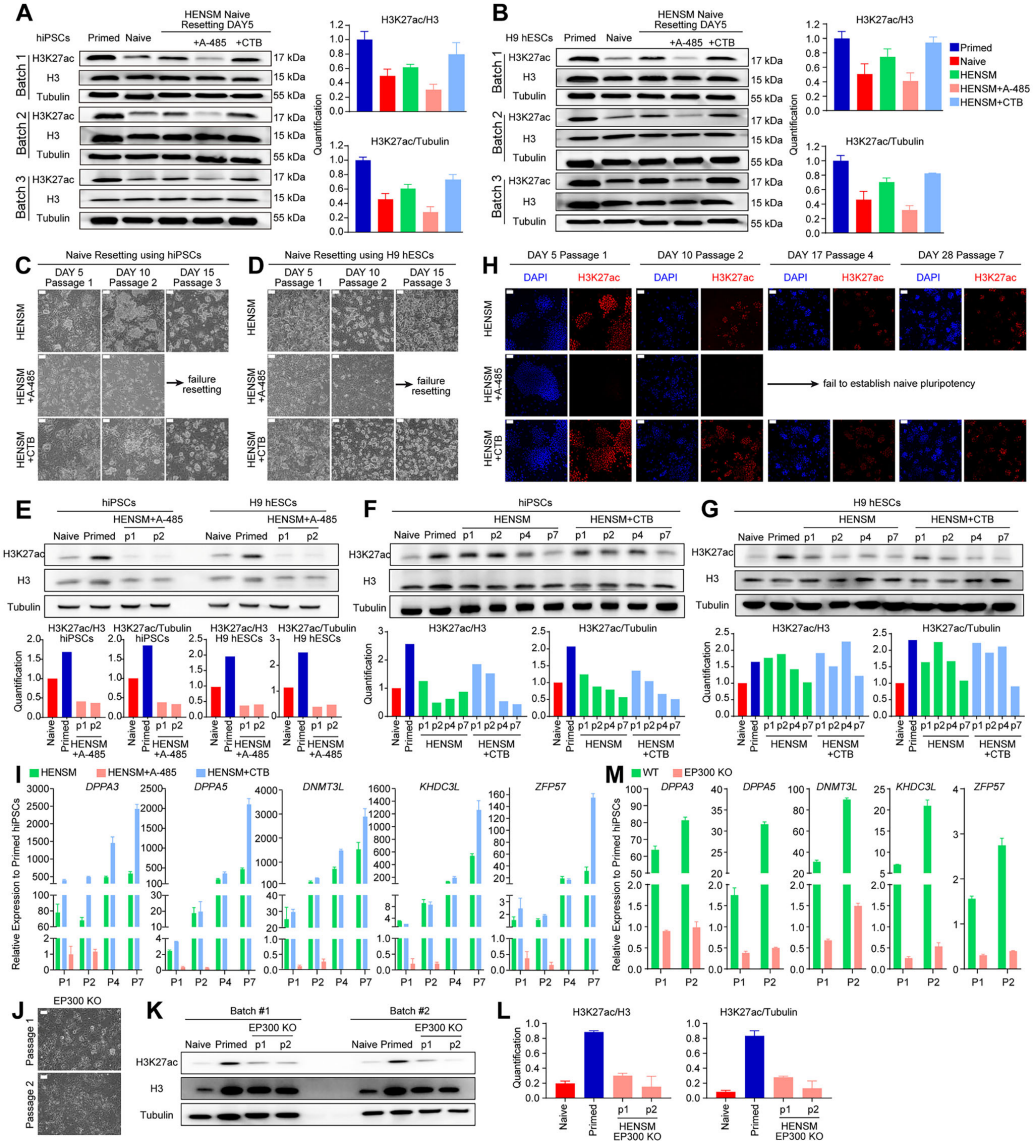
H3K27ac regulation in the establishment of human naive pluripotency. A,B) Western blot analysis showing the H3K27ac levels in primed and naive hiPSCs and H9 hESCs, and HENSM resetting cells treated with EP300 inhibitors (A‐485) or activators (CTB). Error bars indicate ± 1 SD of technical replicates. C,D) Representative images of HENSM resetting hiPSCs or H9 hESCs treated with EP300 inhibitors (A‐485) or activators (CTB) on day 5, 10, and 15 (three passages). Scale bars indicate 200 µm. Western blot analysis showing H3K27ac levels during the early phase of HENSM naive resetting under E) A‐485 and F–G) CTB treatment. H) Representative images of the resetting cells detecting the H3K27ac levels by IF staining. Scale bars indicate 100 µm. I) Real‐time quantitative gene expression analysis for *DPPA3*, *DPPA5*, *DNMT3L*, *KHDC3L*, and *ZFP57* in the resetting cells. Error bars indicate ± 1 SD of technical replicates. J) Representative images of the *EP300* KO cells at P1 and P2 of HENSM naive resetting. Scale bars indicate 200 µm. K) Western blot analysis showing H3K27ac levels in *EP300* KO naive resetting cells at P1 and P2. L,K) Quantification of H3K27ac levels with H3 (left panel) and Tubulin (right panel) based on the western blot results. Error bars indicate ± 1 SD of technical replicates. M) RT‐qPCR analysis for *DPPA3*, *DPPA5*, *DNMT3L*, *KHDC3L*, and *ZFP57* in the *EP300* KO naive resetting cells.

During early naive resetting within 5 d, H3K27ac levels decreased rapidly (**Figure** **4**A,B). Adding two small molecule compounds with opposite effects effectively regulated H3K27ac levels. Specifically, A‐485 reduced H3K27ac levels, while CTB slowed down the decreasing H3K27ac level, which was close to the level of the initial primed hiPSCs (Figure 4A,B). Importantly, after H3K27ac inhibition, primed hiPSCs or H9 hESCs failed to form dome‐like colonies and rapidly differentiated (Figure 4C,D), whereas maintained normal cell proliferation (Figure S4E, Supporting Information). Conversely, activating H3K27ac successfully induced naive‐like colonies (Figure 4C,D). Upon A‐485 addition, H3K27ac levels rapidly decreased during naive resetting (Figure 4E). Previous studies demonstrated that A‐485 only inhibits H3K27ac and H3K18ac modifications.^[^ ^25^ ^]^ To confirm the specific role of H3K27ac modification during naive resetting, we tested the H3K27ac and H3K18ac levels of resetting cells under A‐485 treatment. No significant differences of H3K18ac levels were observed between naive and primed hiPSCs, and also in the resetting cells (Figure S4G,H, Supporting Information). Since SIRT7 protein effectively inhibits H3K18ac modification,^[^ ^26^ ^]^ we performed the HENSM resetting with addition of SIRT7 protein. Dome‐shaped colonies were observed (Figure S4I, Supporting Information) with high alkaline phosphatase activity (Figure S4J, Supporting Information) and increasing expression levels of naive pluripotent genes (Figure S4K, Supporting Information). These results indicate that the addition of A‐485 only inhibits H3K27ac medication during resetting and dampens the establishment of naïve pluripotency.

Without any treatment, H3K27ac levels were initially high but gradually decreased as the naive resetting progressed (Figure 4F,G and Figure S4L,M, Supporting Information). This aligns with previous findings that the expression levels of acetylated H3 histone at lysine 27 was higher in primed hiPSCs than in naive hiPSCs (Figure 2D). Following CTB addition, H3K27ac levels slightly slow down (Figure 4F,G). IF staining analysis of H3K27ac levels yielded similar patterns with the western blot results (Figure 4H). The levels of EP300 protein were not affected under A‐485 or CTB treatment during resetting (Figure S4F, Supporting Information). This is because A‐485 inhibits the function of EP300 protein by competing with acetyl‐CoA.^[^ ^25^ ^]^ qRT‐PCR results showed that A‐485 treatment sharply downregulated naive pluripotent genes within 10 d (two passages) (Figure 4I), indicating these genes are primarily regulated by H3K27ac modification during early naive pluripotency establishment. However, enhancing H3K27ac modification through CTB upregulated naive pluripotency genes, with DPPA3 significantly upregulated by day 5 (Figure 4I), consistent with the previous finding that DPPA3 is essential for establishing human naive pluripotency.^[^ ^21^ ^]^

Furthermore, we established EP300 knock‐out hiPSC lines. This EP300 KO primed hiPSCs dramatically decreased the protein level of EP300 (Figure S4N, Supporting Information). When resetting to P2, similar differentiation with A‐485 treatment was observed (Figure 4J) with the decreased H3K27ac level (Figure 4K,L and Figure S4O, Supporting Information). Taken together, these findings suggest that EP300‐mediated H3K27ac modification is essential during the early stages of human naive pluripotency establishment.

### 2.5 H3K27ac Activation Accelerates the Expression of Naive‐Specific Genes

**Figure 5:**
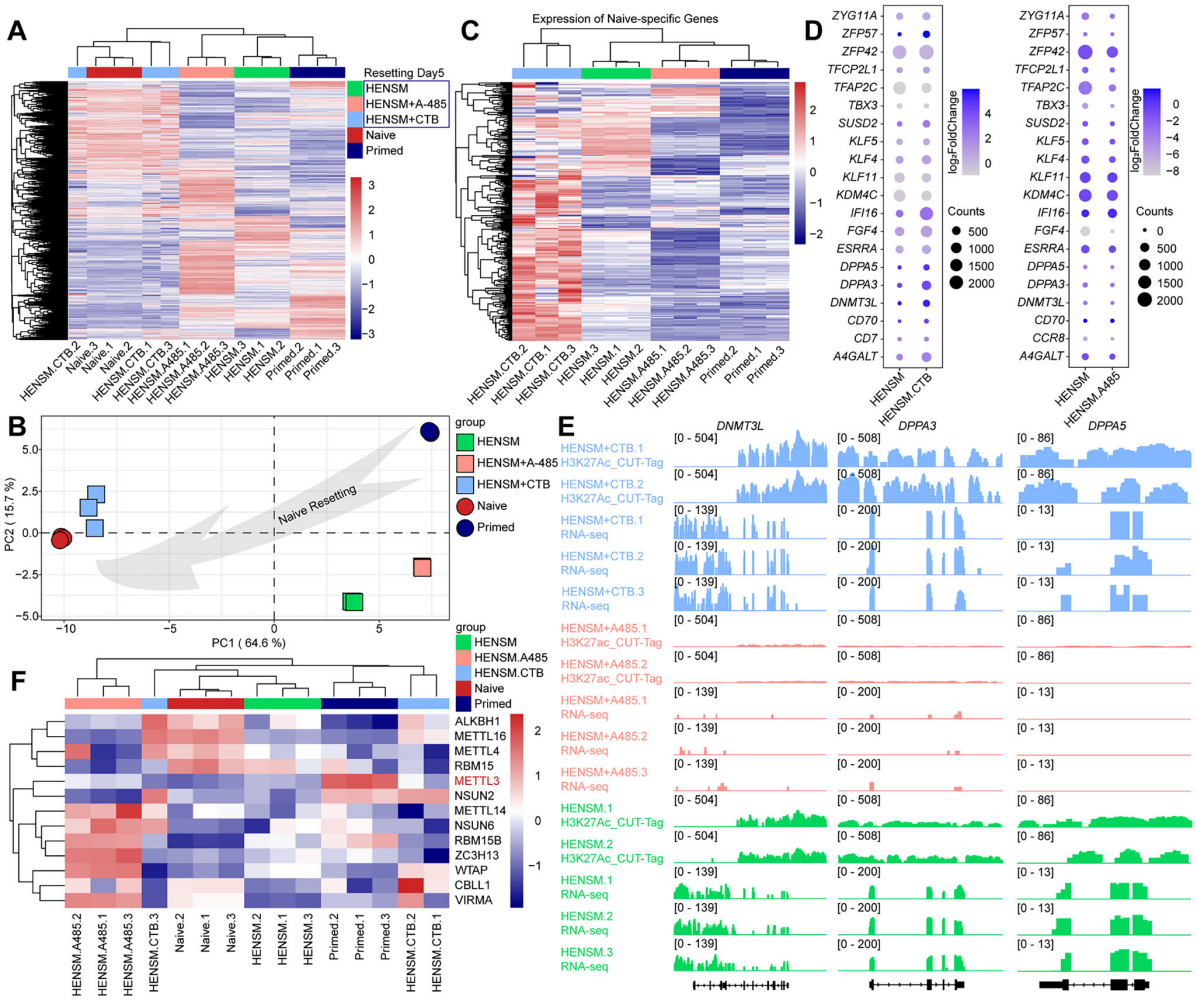
H3K27ac accelerates the expression of naive‐specific genes. A,B) Hierarchical clustering heatmap and PCA showing the differential gene expression patterns in HENSM resetting cells with or without A‐485 or CTB treatment on day 5, compared to HENSM naive hiPSCs and primed hiPSCs. C,D) Heatmap and bubble plots displaying the expression of naive‐specific genes in the HENSM resetting cells. E) IGV visualization showing the differential H3K27ac peaks of modification (CUT&Tag sequencing analysis) and expression (RNA‐seq analysis) in naive pluripotent genes (*DNMT3L*, *DPPA3*, *DPPA5*).

To further elucidate H3K27ac's role in establishing human naive pluripotency, we conducted RNA‐seq on resetting cells on day 5 with or without A‐485 or CTB treatment. Hierarchical clustering analysis revealed significant changes in gene expression patterns after A‐485 inhibited H3K27ac (**Figure** **5**A). The gene expression pattern of CTB‐activated H3K27ac cells closely resembled established naive hiPSCs (Figure 5A). PCA showed that after only 5 d of CTB‐enhanced H3K27ac activation, cells were very similar to the established naive hiPSCs (Figure 5B). In contrast, inhibiting H3K27ac significantly delayed the establishment of naive pluripotency (Figure 5B). Numerous differentially expressed genes were observed between the resetting cells with H3K27ac inhibition or activation and those without small molecule treatments (Figure S5A, Supporting Information).

Next, we analyzed the expression levels of naive‐specific upregulated genes from the integrated analysis and found that CTB‐activated H3K27ac significantly increased their expression. Conversely, inhibiting H3K27ac also suppressed the expression of many naive‐specific upregulated genes (Figure 5C,D and Figure S5B, Supporting Information). Notably, compared to conventionally resetting cells, CTB‐activated H3K27ac upregulated nearly all naive‐specific genes early (Figure 5C,D and Figure S5B, Supporting Information), further suggesting H3K27ac's critical role in the early stages of establishing human naive pluripotency. To verify if naive pluripotency genes are directly regulated by H3K27ac, we performed H3K27ac CUT&Tag sequencing on treated and untreated cells during early resetting. CTB‐activated H3K27ac cells exhibited significant enrichment of H3K27ac peaks in three defined naive pluripotency genes: *DNMT3L*, *DPPA3*, and *DPPA5* (Figure 5E). In contrast, A‐485 inhibition of H3K27ac resulted in almost undetectable H3K27ac peaks and a lack of gene expression (Figure 5E). These findings indicate that in early stages of establishing human naive pluripotency, H3K27ac directly modifies naive pluripotency genes to activate transcription, confirming its essential role in this process.

To explore epigenetic differences, we identified multiple epigenetic writers and erasers from the Epifactor database (https://epifactors.autosome.org/) (Figure S5C,D, Supporting Information) and examined their expression levels in cells treated with A‐485 or CTB. CTB treated resetting cells were closer to the established naive hiPSCs on the expression patterns of genes related to DNA methylation, histone acetylation, methylation, phosphorylation, and ubiquitination, compared to the conventionally HENSM resetting cells (Figure S5C, Supporting Information). Notably, RNA methylation related gene expression patterns differed from other epigenetic modification genes between conventionally HENSM and CTB treated resetting cells, showing no distinct clustering (Figure 5F and Figure S5C, Supporting Information). This suggests that RNA methylation is not affected by H3K27ac during human naive pluripotency establishment and may represent an upstream regulatory mechanism.

### 2.6 m6A Modification Regulates Histone Acetylation and Human Naive Pluripotency

**Figure 6:**
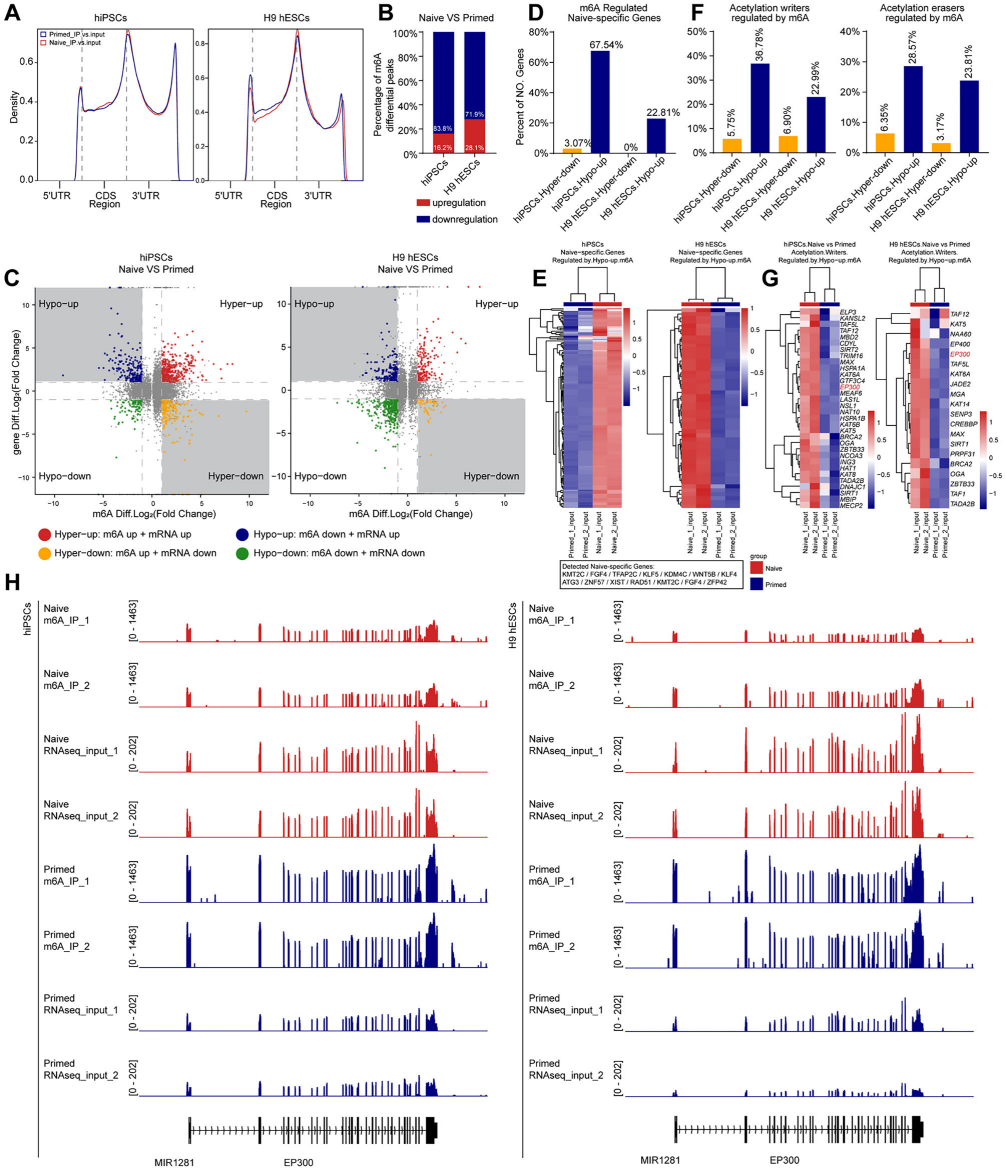
m6A modification regulates histone acetylation and pluripotency gene expression. A) The distribution of m6A peaks in 5′UTR, CDS, and 3′UTR regions in naive and primed hiPSCs (left panel) or H9 hESCs (right panel). B) Histograms showing the percentage of m6A peak numbers between naive and primed hiPSCs or H9 hESCs. C) Classification of m6A‐modified genes into four categories: m6A hypomethylation and upregulated gene expression (Hypo‐up), m6A hypermethylation and upregulated gene expression (Hyper‐up), m6A hypomethylation and downregulated gene expression (Hypo‐down), and m6A hypermethylation and downregulated gene expression (Hyper‐down). D) Histogram showing the percentage of naive‐specific gene numbers in each category. E) Heatmaps showing the expression of naive‐specific genes in Hypo‐up category. F) Histogram showing the percentage of acetylation writer gene numbers in each category. G) Heatmaps showing the expression of acetylation writer genes in Hypo‐up category. H) m6A peaks of modification (MeRIP‐seq analysis) and expression (RNA‐seq analysis) between naive and primed hiPSCs.

To test this hypothesis, we conducted MeRIP‐seq to detect m6A in primed and naive pluripotent stem cells. Analysis showed that m6A enrichment peaks in both primed and naive pluripotent stem cells were concentrated in CDS and 3′UTR, with no significant differences between them (**Figure** **6**A). The total length of m6A peaks enriched by MeRIP‐seq was also similar across different pluripotent stem cells (Figure S6A, Supporting Information). However, analysis of differentially modified peaks showed that naive pluripotent stem cells largely downregulated m6A modification (Figure 6B). These downregulated m6A peaks were widely distributed across 5′UTR, 3′UTR, exons (Figure S6B, Supporting Information), and throughout chromosomes (Figure S6C, Supporting Information). As an RNA‐level inhibitory modification, m6A shows lower levels in naive pluripotent stem cells, suggesting higher transcriptional activity compared to primed pluripotent stem cells.

Based on m6A modification and RNA expression levels, we categorized genes into four groups: Hypo‐up (downregulated m6A, upregulated expression), Hypo‐down (downregulated m6A, downregulated expression), Hyper‐up (upregulated m6A, upregulated expression), and Hyper‐down (upregulated m6A, downregulated expression) (Figure 6C,D and Figure S6D, Supporting Information). Many naive‐specific genes upregulated within the Hypo‐up category (Figure 6D), with significantly higher expression in naive hiPSCs compared to primed hiPSCs (Figure 6E). This suggests that naive hiPSCs downregulate m6A modification to ensure naive‐specific gene expression. Compared to Hyper‐down genes, Hypo‐up genes include many histone acetylation writers and erasers (Figure 6F), with significantly higher expression in naive hiPSCs than in primed hESCs (Figure 6G). This suggests that naive pluripotent stem cells downregulate m6A on regulatory genes to ensure histone acetylation activity. For H3K27ac modification, EP300 expression is significantly higher in naive than in primed pluripotent stem cells, while m6A modification of EP300 is downregulated in naive cells (Figure 6H). This suggests that naive pluripotent stem cells reduce m6A's effect on EP300 to maintain EP300‐mediated H3K27ac modification.

### 2.7 METTL3‐Mediated EP300 Regulates H3K27AC to Facilitate the Establishment of Human Naive Pluripotency

**Figure 7:**
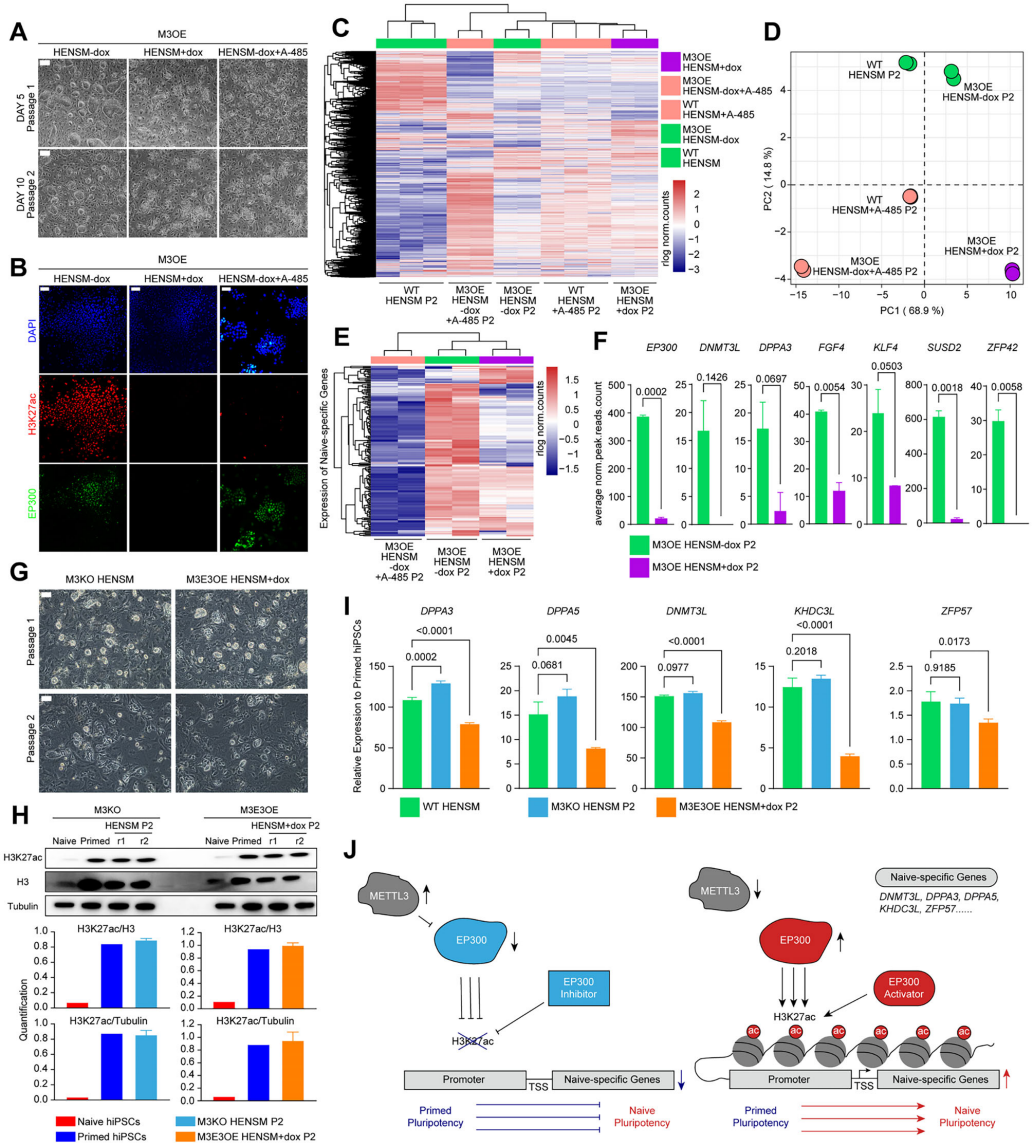
METTL3‐mediated regulation of EP300 and H3K27ac in human naive pluripotency. A) Representative images of M3OE resetting cells treated without dox, with dox, and without dox and with A‐485 at day 5 (P1) and day 10 (P2). Scale bar indicates 200 µm. B) Representative images of M3OE resetting cells detecting H3K27ac and EP300 by IF co‐staining. Scale bar indicates 100 µm. C,D) Hierarchical clustering heatmap and PCA showing the differential gene expression patterns in M3OE resetting cells with or without dox or A‐485 treatment on day 5, compared to WT HENSM resetting cells with or without A‐485 treatment. E) Heatmap showing the expression of naive‐specific genes in M3OE resetting cells. F) Histograms displaying the average normalized H3K27ac counts of EP300 and naive pluripotent genes (*DNMT3L*, D*PPA3*, *FGF4*, *KLF4*, *SUSD2*, and *ZFP42*) in M3OE resetting cells with or without dox treatment. Error bars indicate ± 1 SD of technical replicates. G) Representative images of M3KO and M3E3OE resetting cells at P1 and P2. Scale bars indicate 200 µm. H) Western blot analysis showing the H3K27ac levels in M3KO and M3E3OE resetting cells, compared to primed and naive hiPSCs. Error bars indicate ± 1 SD of technical replicates. I) Real‐time quantitative gene expression analysis for *DPPA3*, *DPPA5*, *DNMT3L*, *KHDC3L*, and *ZFP57* in the M3KO and M3E3OE resetting cells, compared to WT resetting cells. Error bars indicate ± 1 SD of technical replicates. J) Model of METTL3‐mediated regulation of EP300 and H3K27ac in naive pluripotency.

To investigate whether m6A modification regulates EP300‐mediated H3K27ac during the early stages of human naive resetting, we established METTL3 (the primary m6A writer gene) knocking‐out and doxycycline induced overexpressed hiPSC lines, called M3KO and M3OE hiPSCs, respectively. The expression level of *METTL3* in M3KO primed hiPSCs significantly downregulated and higher level of *METTL3* in dox+ M3OE primed hiPSCs was detected (Figure S7A, Supporting Information). The m6A levels were regulated by changes in *METTL3* expression levels (Figure S7B, Supporting Information). Notably, the expression level of *EP300* in these cells exhibited no significant differences (Figure S7A, Supporting Information), indicating that m6A modification does not influence the mRNA level of *EP300*. During naive resetting, dox+ M3OE hiPSCs showed morphology similar to A‐485‐treated resetting cells, and neither could form naive‐like colonies (**Figure** **7**A). The H3K27ac levels were downregulated both in *METTL3* overexpressed and A‐485 treated resetting cells (Figure 7B). Dox+ M3OE resetting cells also downregulated EP300 protein level (Figure 7B), suggesting that m6A modification can inhibit the translation of EP300 protein and further influence the H3K27ac modification during human naive resetting.

Next, we performed RNA‐seq on dox+ and dox‐ M3OE resetting cells, finding significant upregulation of *METTL3* under doxycycline induction (Figure S7C, Supporting Information). Hierarchical clustering revealed that dox+ M3OE resetting cells clustered with A‐485‐treated cells (Figure 7C). Correlation coefficient analysis showed similar results (Figure S7D, Supporting Information). PCA indicated that dox+ M3OE resetting cells were similar to A‐485‐treated cells, but distinct from conventionally resetting cells (Figure 7D). Through the differential gene expression analysis (Figure S7E, Supporting Information), we detected naive‐specific gene expression in these cells. Unlike A‐485‐treated cells inhibiting almost all naive‐specific genes, dox+ M3OE resetting cells inhibited more than half of these genes (Figure 7E; Figure S7E,F, Supporting Information), further indicating that m6A upregulation and H3K27ac inhibition yield similar phenotypes during naive resetting. Enhanced m6A modification significantly inhibited the genome‐wide H3K27ac modification (Figure S7G,H, Supporting Information), especially on EP300 and naive pluripotent genes (Figure 7F).

Furthermore, we wonder whether knocking out METTL3 or overexpressing *EP300* in M3OE hiPSCs could rescue the H3K27ac modification. We established *METTL3* and *EP300* double dox‐inducing overexpression hiPSC line, termed as M3E3OE hiPSCs. With doxycycline induction, M3E3OE primed hiPSCs highly expressed *METTL3* and *EP300* (Figure S7A, Supporting Information). During naive resetting, M3KO and dox+ M3E3OE cells slowed down the differentiation at P2 and even formed few dome‐shaped colonies (Figure 7G). The H3K27ac levels were recovered in M3KO and dox+ M3E3OE resetting cells (Figure 7H and Figure S7I, Supporting Information). The expression levels of naive pluripotent genes in M3KO resetting cells were upregulated to the levels of conventional resetting cells, whereas dox+ M3E3OE resetting cells only recovered partially (Figure 7I).

Taken together, these findings indicate that *METTL3* inhibits EP300 protein translation via m6A upregulation, similar to the effects of direct EP300 inhibition, both leading to H3K27ac inhibition. The absence of H3K27ac modification prevents human naive pluripotency establishment. Conversely, increasing EP300 expression and enhancing H3K27ac modification accelerated human naive pluripotency establishment (Figure 7J).

## 3 Discussion

Histone modifications, including methylation, acetylation, ubiquitination, and phosphorylation, are essential for regulating gene expression during pluripotency and early embryonic development.^[^ ^27^ ^]^ Methylation of histone H3 at lysine 27 (H3K27me3) and lysine 4 (H3K4me3) is well‐studied; H3K27me3 marks repressed genomic regions, while H3K4me3 marks active promoters.^[^ ^13^, ^28^ ^]^ Histone acetylation, particularly H3K27ac, promotes transcription by facilitating an open chromatin conformation.^[^ ^14^, ^29^ ^]^ These histone marks are dynamically regulated during transitions between naive and primed pluripotency in mouse and human models.^[^ ^15^, ^30^ ^]^ Ubiquitination, another post‐translational modification, influences chromatin structure and gene expression, especially in DNA repair and histone degradation.^[^ ^31^ ^]^ Though less well‐studied, histone phosphorylation impacts chromatin compaction and transcriptional regulation during cellular differentiation.^[^ ^32^ ^]^ These modifications collectively fine‐tune gene expression, allowing pluripotent stem cells to maintain their identity while remaining poised for differentiation.^[^ ^13^, ^33^ ^]^ Our findings demonstrate that these epigenetic modifications are essential for establishment and maintaining pluripotency. In addition, we found that histone modifications, particularly H3K27ac, are differentially enriched between naive and primed states, suggesting a key role in pluripotency transitions.

H3K27ac, a critical histone acetylation mark, is predominantly associated with active enhancers and promoters, regulating gene expression during pluripotency and early development.^[^ ^13^, ^34^ ^]^ In our study, H3K27ac modification was significantly more abundance in naive hiPSCs compared to primed cells (Figures 3 and 4). This enrichment suggests that H3K27ac is essential for maintaining the open chromatin state required for naive pluripotency.^[^ ^35^ ^]^ The acetyltransferase EP300, which catalyzes H3K27 acetylation, is critical for this process, ensuring that key pluripotency genes remain accessible for transcription.^[^ ^7^, ^14^, ^15^, ^36^ ^]^ Our findings align with previous studies, indicating that H3K27ac sustains the naive state, potentially by enhancing pluripotency‐associated enhancer activity.^[^ ^37^ ^]^ However, the mechanisms by which H3K27ac influence pluripotent state transitions, particularly in human cells, remain unclear. Future studies should identify specific enhancer regions modulated by H3K27ac and examine their interactions with other epigenetic modifications during state transitions.

m6A is a critical gene expression regulator in pluripotent stem cells, influencing mRNA stability and translation efficiency.^[^ ^16^, ^18^ ^]^ Our study showed differential m6A patterns between naive and primed states, with naive cells exhibiting higher m6A levels at key pluripotency‐associated transcripts (Figure 6). This supports the role of m6A in maintaining the naive state by promoting differentiation‐related transcript degradation and stabilizing pluripotency factors.^[^ ^17^, ^19^ ^]^ METTL3, the enzyme catalyzing m6A methylation, is essential for this process, ensuring proper RNA metabolism and pluripotency maintenance.^[^ ^17^, ^18^, ^19^, ^38^ ^]^ Consistent with previous studies, our data suggest that m6A modifications fine‐tune the balance between self‐renewal and differentiation in human pluripotent stem cells.^[^ ^39^ ^]^ However, the molecular mechanisms by which m6A regulates pluripotent state transitions, particularly its interplay with other epigenetic modifications like histone acetylation, remain open for investigation.

Epigenetic modifications, including m6A, H3K27ac, and DNA methylation, collectively regulate pluripotency and early embryonic development.^[^ ^12^, ^36^ ^]^ The interaction between m6A and H3K27ac is intriguing, as m6A likely influences histone modification patterns, including acetylation, by modulating the expression and stability of chromatin‐modifying enzymes.^[^ ^40^ ^]^ Our results suggest an interplay between these modifications, with m6A potentially acting upstream of H3K27ac in regulating pluripotency gene expression.^[^ ^41^ ^]^ The dynamic regulation of these modifications is crucial for maintaining pluripotency, as both m6A and H3K27ac promote open chromatin and enhance pluripotency gene transcription. Although progress has been made in understanding individual epigenetic modifications, their combined regulatory effects on human pluripotency and early development remain poorly understood.^[^ ^42^ ^]^ Future studies should map genome‐wide interactions between these modifications and elucidate how they cooperate to regulate transitions between naive and primed states.

Understanding the interplay between m6A, H3K27ac, and DNA methylation provides insights into regulating early human embryonic development and pluripotency.^[^ ^19^, ^43^ ^]^ Our study highlights the potential of these modifications to control gene expression and cell fate, indicating their role in establishing primitive pluripotent states, such as totipotent stem cells.^[^ ^1a^ ^]^ Further dissecting how these modifications regulate transcriptional programs may uncover strategies to reprogram human cells to earlier, more potent developmental states.^[^ ^40^ ^]^ This knowledge may enable the generation of cells with enhanced developmental plasticity, offering valuable applications in regenerative medicine and disease modeling.^[^ ^19^, ^43^ ^]^ However, significant challenges remain, including understanding the precise temporal dynamics and interactions of these epigenetic marks during transitions between naive, primed, and totipotent states. Future efforts should integrate multi‐omics approaches to elucidate these regulatory networks, paving the way for developing more robust stem cell models.

### 3.1 Limitations of the Study

This study revealed an inverse relationship between total H3K27ac‐modified histone abundance and modification breadth across pluripotent states: naive hiPSCs exhibited reduced global histone levels yet broader H3K27ac genomic distribution, whereas primed hiPSCs displayed elevated histone content but spatially restricted modifications. ChIP‐seq analyses demonstrated that naive hiPSCs had extensive but low‐density H3K27ac modifications (characterized by average peak reads and average peak fold enrichment), while primed hiPSCs showed focal hyper‐dense modifications at limited genomic loci (Figure 3H,I). Although these observations provide insight into the increased total histone content in primed hiPSCs, key questions remain unresolved: (1) whether this inverse H3K27ac abundance–distribution relationship represents a universal feature of pluripotency states; (2) whether genomic loci harboring hyper‐dense modifications in primed pluripotency possess unique functional properties. Future studies should validate these findings across diverse hPSC lines and naive hPSCs derived through alternative resetting methods.

## 4 Experimental Section

### Culturing of HENSM Naive hPSCs

For the naive culture of HENSM, 2 × 10^6^ primed hESCs were initially seeded atop a mouse embryonic fibroblast (MEF) feeder layer, utilizing E8 medium with 5 × 10^−6^ m Y‐27632. After 24 h, the medium was replaced with the HENSM medium.^[^ ^4l^ ^]^ The naive cells were stably passaged using TrypLE for dissociation every 3–5 d. A total of 500 mL of HENSM medium was prepared by combining 240 mL Neurobasal, 240 mL DMEM/F12, 5 mL N2 supplement, 10 mL B27 supplement, 5 mL GlutaMAX, 1% nonessential amino acids, and 1% Penicillin‐Streptomycin, supplemented with 0.8 × 10^−3^ m dimethyl 2‐oxoglutarate (aKG), 1 × 10^−3^ m PD0325901, 2 × 10^−3^ m XAV939, 2 × 10^−3^ m Gö6983, 1.2 × 10^−3^ m CGP77675, 20 ng mL^−1^ human LIF, and 20 ng mL^−1^ Activin A. All resetting experiments in this paper were performed under 5% O_2_ and 5% CO_2_.

### Culturing of hEPSCs

To obtain hEPSCs, 5 × 10^5^ primed hESCs were seeded on the feeder layer in E8 medium supplemented with 10 × 10^−6^ m Y‐27632. On the second day, the medium was switched to N2B27‐LCDM medium.^[^ ^5b^ ^]^ A total of 500 mL of LCDM medium was prepared by combining 240 mL DMEM/F12, 240 mL Neurobasal, 2.5 mL N2 supplement, 5 mL B27 supplement, 1% GlutaMAX, 1% nonessential amino acids, 0.1 × 10^−3^ m β‐mercaptoethanol, penicillin‐streptomycin, and either 5 mg mL^−1^ BSA or 5% knockout serum replacement, supplemented with 10 ng mL^−1^ recombinant human LIF, 1 × 10^−6^ m CHIR99021, 2 × 10^−6^ m (S)‐(+)‐dimethindene maleate, 2 × 10^−6^ m minocycline hydrochloride, 1 × 10^−6^ m IWR‐endo‐1, and 2 × 10^−6^ m Y‐27632. Cells were passaged every 3 d using Accutase.

### Overexpression of METTL3

Lentiviruses were generated in 293 cells. Culture medium was changed 12 h post‐transfection and virus‐containing supernatant was collected 48–72 h post‐transfection. The viral supernatant was filtered through a 0.45 mm filter. Virus‐containing supernatants of FUW‐tetO‐METTL3 supplemented with the FUW‐M2rtTA virus and an equal volume of fresh culture medium. 1 × 10^6^ hESCs were seeded 24 h before transduction in T75 flasks on Matrigel in E8 medium. The infections in the presence of 2 mg mL^−1^ of polybrene were performed over a period of 24 h. Culture medium was changed 12 h after infection.

### Protein Extraction

The sample was subjected to three rounds of sonication on ice using a high‐intensity ultrasonic processor (Scientz) in a lysis buffer containing 8 m urea, 1% protease, 3 × 10^−6^ m TSA, and 50 × 10^−3^ m NAM. Subsequently, residual debris was eliminated through centrifugation at 12 000 *g* at 4 °C in 10 min. The supernatant was then collected, and protein concentration was quantified using a BCA kit, following the manufacturer's guidelines.

### TMT/iTRAQ Labeling

The protein solution was initially reduced using 5 × 10^−3^ m dithiothreitol for 30 min at 56 °C and subsequently alkylated with 11 × 10^−3^ m iodoacetamide for 15 min at ambient temperature in the absence of light. Following this, the protein sample was diluted by the addition of 100 × 10^−3^ m TEAB to achieve a urea concentration of less than 2 m. Digestion was performed by adding trypsin in a 1:50 trypsin‐to‐protein mass ratio for an overnight incubation, followed by a second digestion using a 1:100 trypsin‐to‐protein mass ratio for 4 h. Post‐trypsin digestion, the peptides were desalted using a Strata X C18 SPE column (Phenomenex) and subsequently vacuum dried. The peptides were then reconstituted in 0.5 m TEAB and prepared according to the manufacturer's instructions for the TMT kit/iTRAQ kit. Specifically, one unit of TMT/iTRAQ reagent was thawed, reconstituted in acetonitrile, and the peptide mixtures were incubated for 2 h at room temperature. Finally, the peptides were pooled, desalted, and dried using vacuum centrifugation.

### HPLC‐MS/MS

After being dissolved in 0.1% formic acid, the peptides were separated using a reverse‐phase analytical column with an internal diameter of 75 µm and a length of 15 cm. The elution process employed a gradient of solvent B, starting from 6% and gradually increasing to 23% over 26 min, then rising to 35% within the next 8 min, swiftly escalating to 80% within the subsequent 3 min, and maintaining this ratio for another 3 min. The flow rate was set at 400 nL min^−1^, utilizing the EASY‐nLC 1000 UPLC system. Mass spectrometry analysis was conducted using a nano electrospray ionization source and the Q Exactive Plus system for MS/MS analysis. The electrospray voltage was set at 2.0 kV, with a scan range of 350–1800 m/z, and the full scan resolution was set at 70 000. For MS/MS analysis, the normalized collision energy was adjusted to 28, with a resolution of 17 500. The experiment employed a data‐dependent acquisition mode, alternating between full scans and 20 MS/MS scans, with a dynamic exclusion time of 15 s and an automatic gain control set at 5E4, with the first mass fixed at 100 m/z.

### ChIP‐seq

Chromatin immunoprecipitation was performed by Wuhan IGENEBOOK Biotechnology. Cells were cross‐linked with formaldehyde, lysed, and chromatins were sonicated. Chromatin was immunoprecipitated with antibodies. After incubation with protein beads, samples were washed and eluted. DNA was treated with RNase A and proteinase K, then used to construct sequencing libraries with the NEXTFLEX ChIP‐Seq Library Prep Kit and sequenced on an Illumina sequencer. FASTQ files underwent quality control with FastQC (0.11.9). Reads were aligned to GRCh38 using BWA (0.7.17), converted to BAM by SAMtools (1.3.1), then sorted and deduplicated using SAMtools and Picard (2.24.0). Peaks were called MACS2 (2.2.7.1) at q‐value 0.05. Regions were defined using UCSC.hg38, with promoters within 3 kb of TSS. Peak annotation was done with ChIPseeker (1.22.1) and org.Hs.eg.db (3.10.0) and visualized using ggplot2 (3.3.2). Motifs were discovered with HOMER (v4.9). BAM files were converted to bigWig with deeptools (3.3.0) and visualized via IGV (2.7.0).

### CUT&Tag

CUT&Tag was conducted following the established CUT&Tag protocol.^[^ ^44^ ^]^ All buffers were enriched with 5 × 10^−3^ m sodium butyrate and 1× complete protease inhibitor. To minimize sample loss, Protein LoBind tubes were utilized. For GAS samples, incubation volumes were doubled to accommodate tissue debris within the nuclear suspensions, resulting in enhanced TapeStation quality control outcomes. Antibodies against H3K27ac were employed. Libraries were indexed using Nextera Indexes, and 150 bp paired‐end sequencing was conducted on an Illumina sequencer.

### RNA‐seq

To prepare RNA for sequencing, one million hPSCs were trypsinized. Total RNA was isolated using Trizol. Sequencing was performed on an Illumina sequencer with 150 bp paired‐end sequencing reaction. All the RNA‐seq data analysis were performed with Hisat2 (version 2.1.0) and Cufflinks (version 2.2.1) using the UCSC human genome annotation (version hg38) with default settings. Reads with unique genome location and genes with no less than 1 FPKM in at least one sample were used for the following analysis.

### MeRIP‐seq

Total RNA was isolated using the Trizol reagent. The RNA quantity and purity were quantified with a NanoDrop ND‐1000 spectrophotometer, and integrity was assessed using a Bioanalyzer 2100 (RIN >7.0) and agarose gel electrophoresis. Poly(A) RNA was extracted from 50 µg of total RNA utilizing Dynabeads Oligo (dT)25‐61005. The poly(A) RNA underwent fragmentation using a Magnesium RNA Fragmentation Module at 86 °C for 7 min. The RNA fragments were then incubated with an m6A‐specific antibody in the IP buffer at 4 °C for 2 h. The immunoprecipitated RNA was reverse‐transcribed into cDNA, followed by the synthesis of U‐labeled second‐strand DNAs. Adapters were ligated to the A‐tailed DNA fragments, with subsequent size selection using AMPure XP beads. The U‐labeled DNAs were treated with UDG enzymes and amplified via PCR. The final cDNA library had an average insert size of 300 ± 50 bp. Finally, 2 × 150 bp paired‐end sequencing was conducted on an Illumina sequencer.

### Immunostaining

For the immunofluorescence experiment, fix the samples with 4% paraformaldehyde (PFA) at room temperature for 10 min. Subsequently, rinse the samples twice with PBST (phosphate‐buffered saline with Triton X‐100). Then, block the samples at room temperature for 1 h with BSA, followed by three washes with PBST. Then immunostaining was performed according to standard protocols using the following primary antibodies: DPPA3 (mouse monoclonal, Abcam); H3K27ac (rabbit monoclonal, PTM BIO); EP300 (rabbit polyclonal, Cell Signaling Technology); appropriate Alexa Fluor dye conjugated secondary antibodies (Invitrogen) were used. Nuclei were stained with DAPI (Life Technologies). Images were taken using Leica TCS Sp8 confocal microscope. Data analysis was performed by ImageJ (1.48 V).

### Western Blot

The whole cell lysates of hPSCs were generated by lysing 5 × 10^6^ washed cells in 200 µL RIPA buffer containing standard protease inhibitors cocktail. Samples were incubated on ice for 10 min and then subjected to BCA analysis. 20 µg of total protein was used for western blot analysis. The following primary and secondary antibodies were used: Anti‐Acetyl‐Histone H3 (Lys27) (PTM BIO), H3 (Cell Signaling Technology), Tubulin (PTM BIO), HRP‐labeled Goat Anti‐Rabbit IgG(H+L) (Beyotime), HRP‐labeled Goat Anti‐Mouse IgG(H+L) (Beyotime).

## Conflict of Interest

The authors declare no conflict of interest.

## Author Contributions

C.A., K.Z., X.K., and Y.Z. contributed equally to this work. Conceptualization, Y.F., Y.Y., and X.S.; Methodology, C.A. and K.Z.; Investigation, C.A., K.Z., and Y.Z.; Writing – original draft, C.A. and K.Z.; Writing – review & editing, C.A., K.Z., L.J., X.K., Y.Y., X.S., and Y.F.; Formal analysis, C.A. and Y.S; Resources, C.A., Y.S., and J.Z.; Validation, K.Z., Y.S., J.Z., H.L., Z.L., and J.L.; Funding acquisition, F.Y., Y.Y., and C.A.; Supervision, F.Y.

## Supporting information



Supporting Information

## Data Availability

The raw sequence data reported in this paper have been deposited in the Genome Sequence Archive (Genomics, Proteomics & Bioinformatics 2021) in National Genomics Data Center (Nucleic Acids Res 2022), China National Center for Bioinformation / Beijing Institute of Genomics, Chinese Academy of Sciences (GSA‐Human: HRA008972) that are publicly accessible at https://ngdc.cncb.ac.cn/gsa‐human. This paper does not report any original code. This study did not generate any unique reagents. Any additional information required to reanalyze the data reported in this paper is available from the lead contact upon request.
