# Supplementary material for: H3K27ac Is Essential for Human Naive Pluripotency Modulated by m6A‐Driven EP300 Expression
Source: Adv Sci (Weinh). 2025 Aug 11;12(41):e07549. doi: 10.1002/advs.202507549 (PMC12591129; doi:10.1002/advs.202507549)
Supplement: Supplementary file 1 — Supporting Information [file ADVS-12-e07549-s001.docx]

**Supplemental Information**

**H3K27ac is Essential for Human Naive Pluripotency Modulated by m6A-Driven EP300 Expression**

*Chenrui An*^1,7^, *Ke Zhong*^1,7^, *Xiangjin Kang*^1,7^, *Yingying Zhang*^1,7^, *Yudan Shang*^1^, *Hao Liu*^1^, *Yushan Ji*^1^, *Jinbei Zeng*^1^, *Jiajin Lin*^1^, *Long Jin*^1^, *Zelong Lin*^1^, *Yang Yu*^2,3,^*, *Xuetao Shi*^4,5,6,^*, *Yong Fan*^1,^*

^1^Department of Obstetrics and Gynecology; Guangdong Provincial Key Laboratory of Major Obstetric Diseases; Guangdong Provincial Clinical Research Center for Obstetrics and Gynecology; Guangdong-Hong Kong-Macao Greater Bay Area Higher Education Joint Laboratory of Maternal-Fetal Medicine; The Third Affiliated Hospital, Guangzhou Medical University, Guangzhou, 510150, China.

^2^Beijing Key Laboratory of Reproductive Endocrinology and Assisted Reproductive Technology and Key Laboratory of Assisted Reproduction, Ministry of Education, Center of Reproductive Medicine, Department of Obstetrics and Gynecology, Stem Cell Research Center, Peking University Third Hospital, Beijing, 100191, China.

^3^Stem Cell Research Center, Peking University Third Hospital, Beijing, 100191, China.

^4^National Engineering Research Centre for Tissue Restoration and Reconstruction and Key Laboratory of Biomedical Engineering of Guangdong Province South China University of Technology Guangzhou 510640, China.

^5^Key Laboratory of Biomedical Engineering of Guangdong Province, South China University of Technology, Guangzhou, 510006, China.

^6^School of Materials Science and Engineering, South China University of Technology, Guangzhou, 510640, China.

^7^These authors contributed equally.

*Correspondence:

yongfan011@gzhmu.edu.cn (Y.F.)

yuyang5012@hotmail.com (Y. Y.)

shxt@scut.edu.cn (X.S.)

**
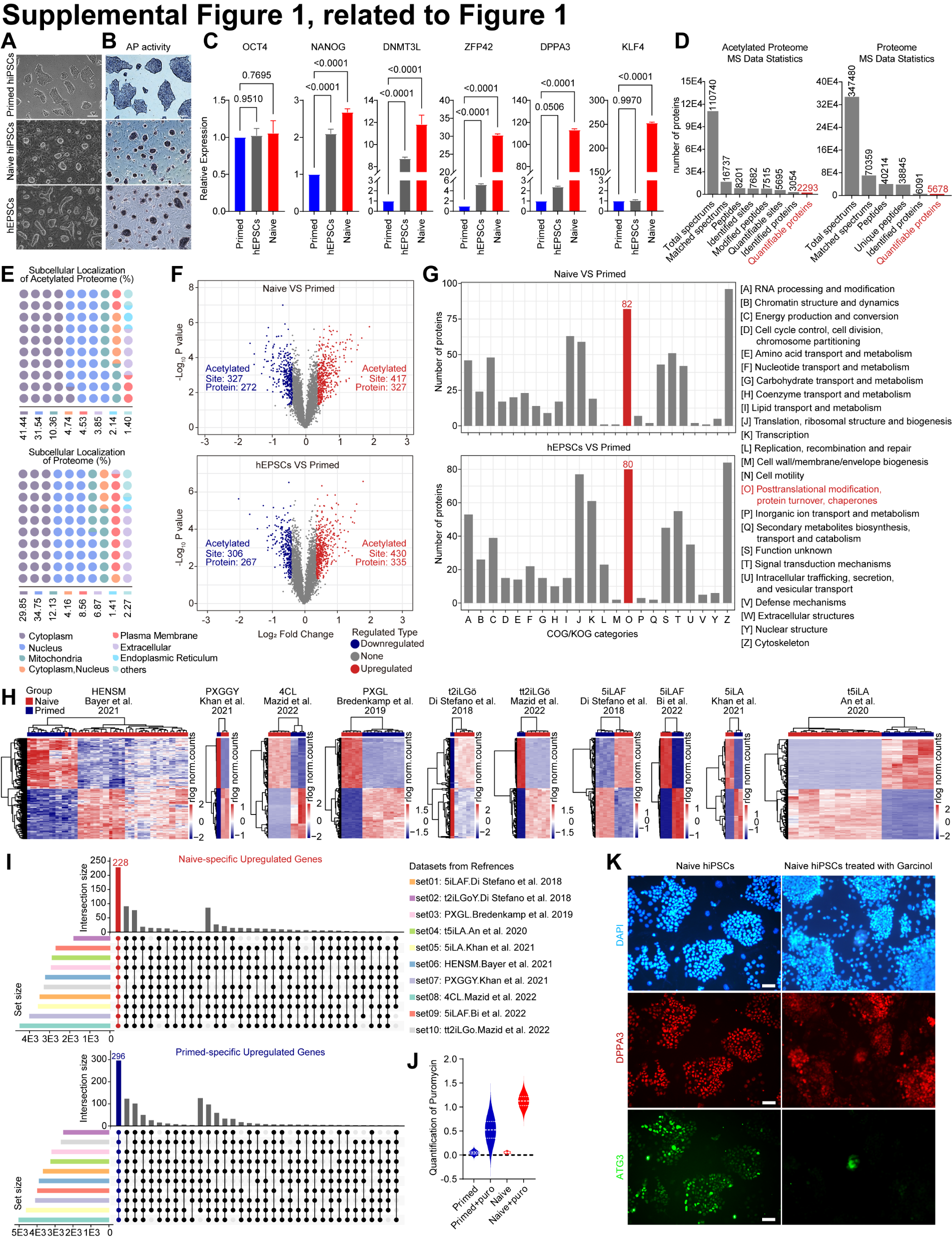
**

*Figure legend on the next page*

**Figure S1**

Proteomic analysis of acetylated proteins in naive and primed hiPSCs, and hEPSCs. [Related to Figure 1].

(**A**) Representative images of primed and naive hiPSCs, and hEPSCs. Scale bars indicate 200 µm. (**B**) Representative images of primed and naïve hiPSCs, and hEPSCs detecting alkaline phosphatase activity. Scale bars indicate 200 µm. (**C**) Real-time quantitative gene expression analysis for *OCT4*, *NANOG*, *DNMT3L*, *ZFP42*, *DPPA3*, and *KLF4*. Error bars indicate ± 1 SD of technical replicates. (**D**) Histograms showing the detected 2,293 quantifiable acetylated proteins and 5,678 total proteins across different pluripotent stem cells. (**E**) Subcellular distribution of acetylation modifications. (**F**) Volcano plots displaying the differentially expressed acetylated proteins between primed and naive hiPSCs, and hEPSCs. X axis represents the differential expression levels using Log_2_ (fold change of expressed acetylated proteins) between samples. Y axis represents the significance between samples using -Log_10_ P value. Red dots represent upregulated proteins, and blue dots represent downregulated proteins. (**G**) COG functional analysis of differential expressed acetylated proteins between samples. (**H-I**) Heatmaps (H) and histograms (I) showing differential gene expression patterns between naive and primed hPSCs, based on the analysis of integrated RNA-seq data from 10 previous studies. (**J**) Quantification of puromycin levels between naive and primed hiPSCs upon the puromycin incorporating. (**K**) Representative images of naive hiPSCs treated with or without Garcinol detecting DPPA3 and ATG3 proteins by IF co-staining. Scale bars indicate 100 µm.

**
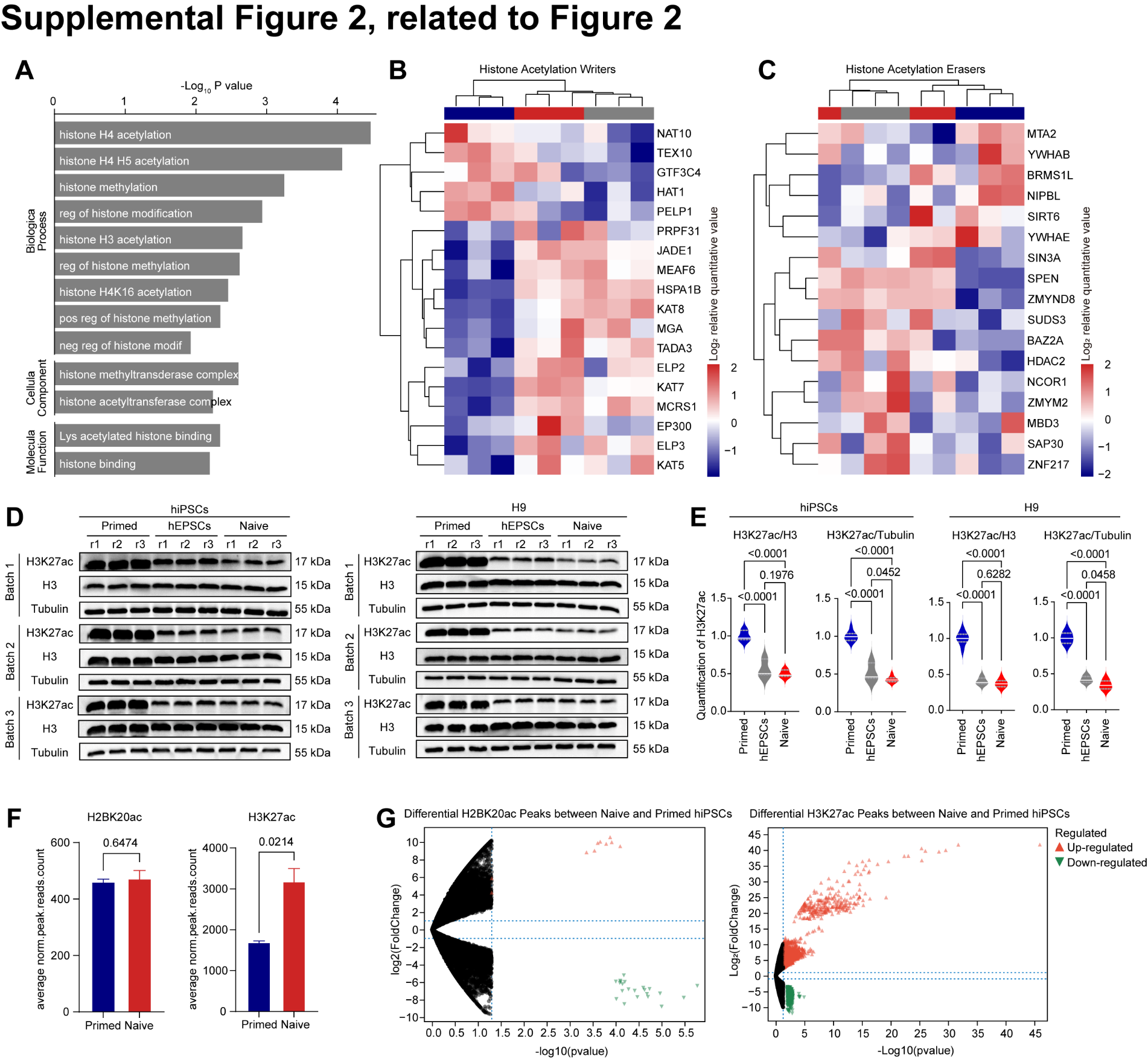
**

*Figure legend on the next page*

**Figure S2**

Histone acetylation and proteomic differences among different pluripotent stem cells. [Related to Fig. 2].

(**A**) GO term and KEGG analysis of differential expressed proteins between naive and primed hiPSCs. (**B-C**) Heatmaps showing the differential expression patterns of histone acetylation writer (B) and eraser (C) gene coding proteins among naive and primed hiPSCs, and hEPSCs. (**D**) Western blot analysis of H3K27ac levels in different pluripotent hiPSCs (left panel) and H9 hESCs (right panel). (**E**) Quantification of H3K27ac levels with H3 or Tubulin based on the western blot results (D). (**F**) Histograms showing the average normalized H2BK20ac (left panel) and H3K27ac (right panel) peak counts in naive and primed hiPSCs. Error bars indicate ± 1 SD of technical replicates. (**G**) Volcano plots displaying the differentially H2BK20ac peaks (left panel) and H3K27ac peaks (right panel) between primed and naive hiPSCs. Y axis represents the differential peak counts using Log_2_ (fold change) between samples. X axis represents the significance between samples using -Log_10_ P value. Red triangles represent upregulated peaks, and blue dots represent downregulated peaks.

**
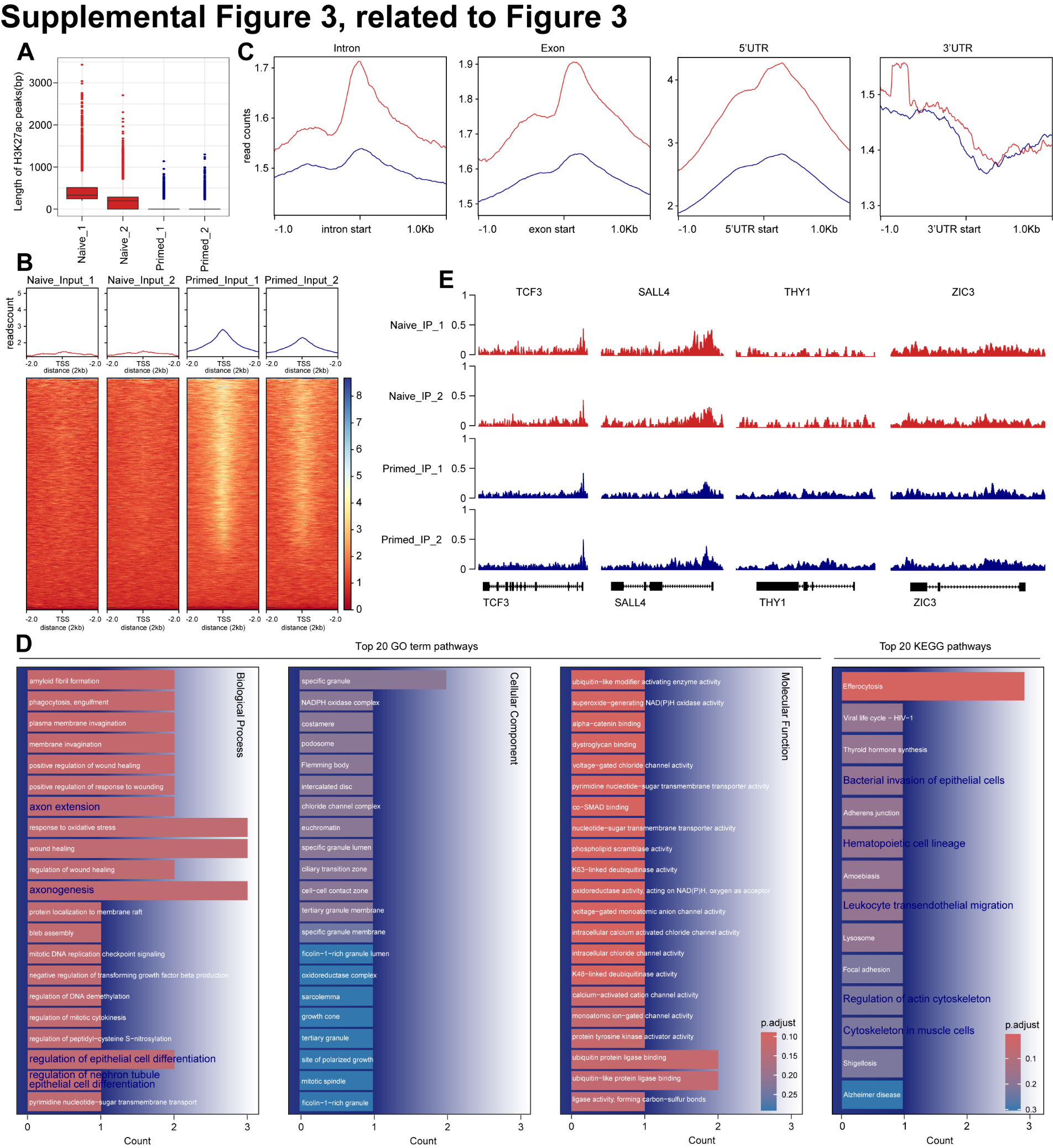
**

*Figure legend on the next page*

**Figure S3**

Genome-wide mapping of H3K27ac in naive and primed hiPSCs. [Related to Fig. 3].

(**A**) Box plots showing the length of H3K27ac peaks in naive and primed hiPSCs. (**B**) Differential enrichment of H3K27ac peaks at TSS in naive and primed hiPSCs. (**C**) Distribution of H3K27ac modification across different genomic regions (exons, introns, promoters, intergenic regions) in naive (red lines) and primed (blue lines) hiPSCs. (**D**) GO term and KEGG analysis of differential H3K27ac-modified genes between naive and primed hiPSCs. (**E**) IGC visualization of H3K27ac peaks on primed-specific genes in naive and primed hiPSCs.

**
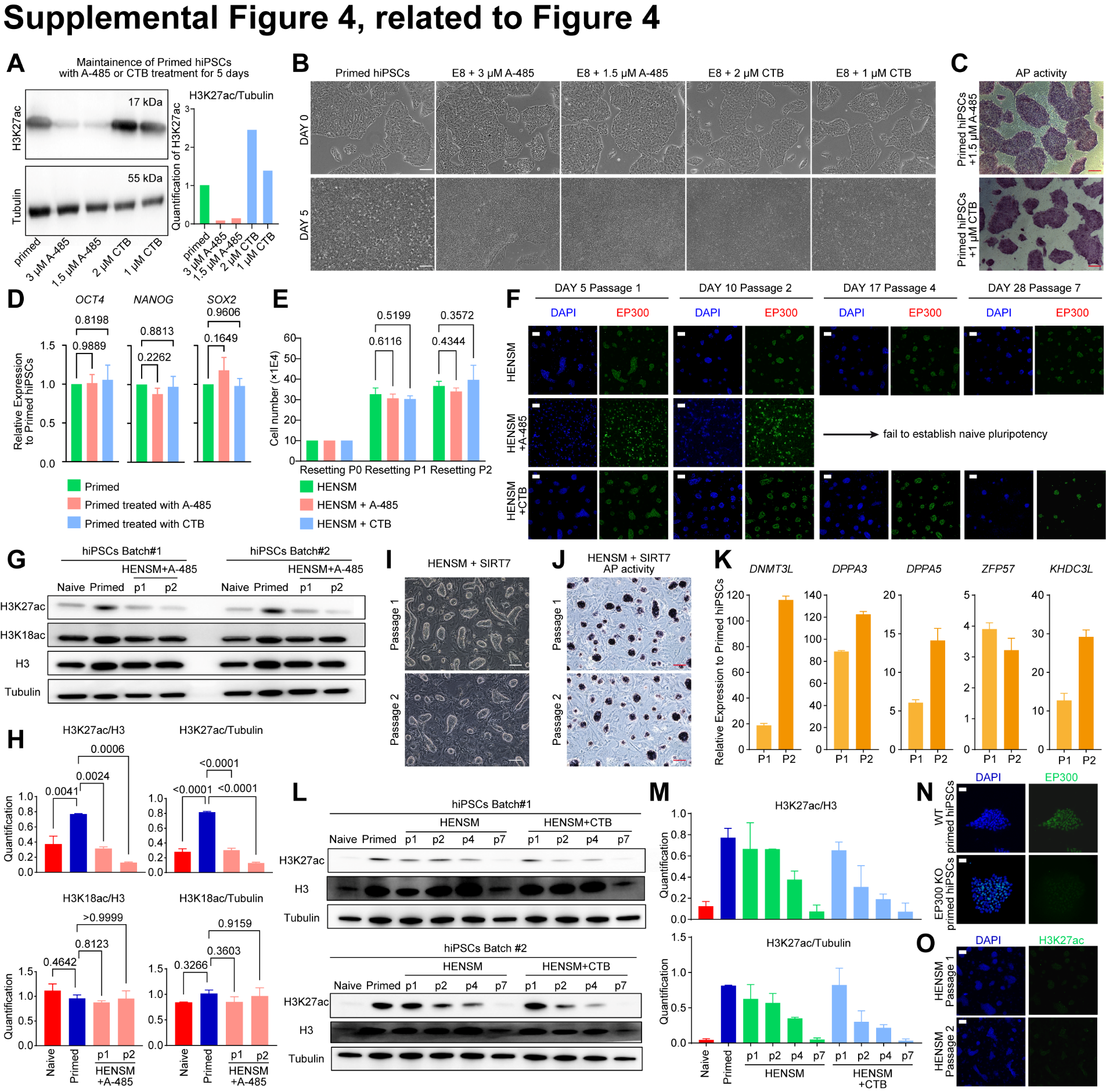
**

*Figure legend on the next page*

**Figure S4**

Functional impact of H3K27ac regulation by small molecules on pluripotency. [Related to Fig. 4].

(**A**) Western blot analysis of H3K27ac levels under different dosages of A-485 or CTB treatment. (**B**) Representative images of primed hiPSCs treated with different dosages of A-485 or CTB at day 0 and day 5. Scales bars indicate 200 µm. (**C**) Representative images of primed hiPSCs treated with 1.5 µM A-485 (upper panel) or 1 µM CTB (lower panel) detecting alkaline phosphatase activity. Scale bars indicate 200 µm. (**D**) RT-qPCR analysis for *OCT4*, *NANOG*, and *SOX2*. Error bars indicate ± 1 SD of technical replicates. (**E**) Histogram showing the cells number of HENSM resetting cells with or without A-485 or CTB from P0 to P2. Error bars indicate ± 1 SD of technical replicates. (**F**) Representative images of HENSM resetting cells with or without A-485 or CTB, detecting EP300 protein using IF staining. Scale bars indicate 100 µm. (**G**) Western blot analysis of H3K27ac and H3K18ac levels in HENSM resetting cells with A-485, compared to HENSM naive hiPSCs and primed hiPSCs. (**H**) Quantification of H3K27ac and H3K18ac levels compared to H3 or Tubulin levels, based on the western blot results in Figure S4G. (**I**) Representative images of HENSM resetting cells treated with SIRT7 proteins. Scale bars indicate 200 µm. (**J**) Representative images of HENSM + SIRT7 resetting cells detecting alkaline phosphatase activity. Scale bars indicate 200 µm. (**K**) RT-qPCR analysis for *DNMT3L*, *DPPA3*, *DPPA5*, *ZFP57*, and *KHDC3L* in HENSM + SIRT7 resetting cells. Error bars indicate ± 1 SD of technical replicates. (**L-M**) Western blot analysis of H3K27ac levels in HENSM resetting cells with CTB, compared to HENSM naive hiPSCs and primed hiPSCs (L), and quantification of H3K27ac levels compared to H3 or Tubulin levels. Error bars indicate ± 1 SD of technical replicates. (**N**) Representative images of WT and EP300 KO primed hiPSCs detecting EP300 proteins by IF staining. Scale bars indicate 100 µm. (**O**) Representative images of EP300 KO HENSM resetting cells detecting H3K27ac by IF staining. Scale bars indicate 100 µm.

**
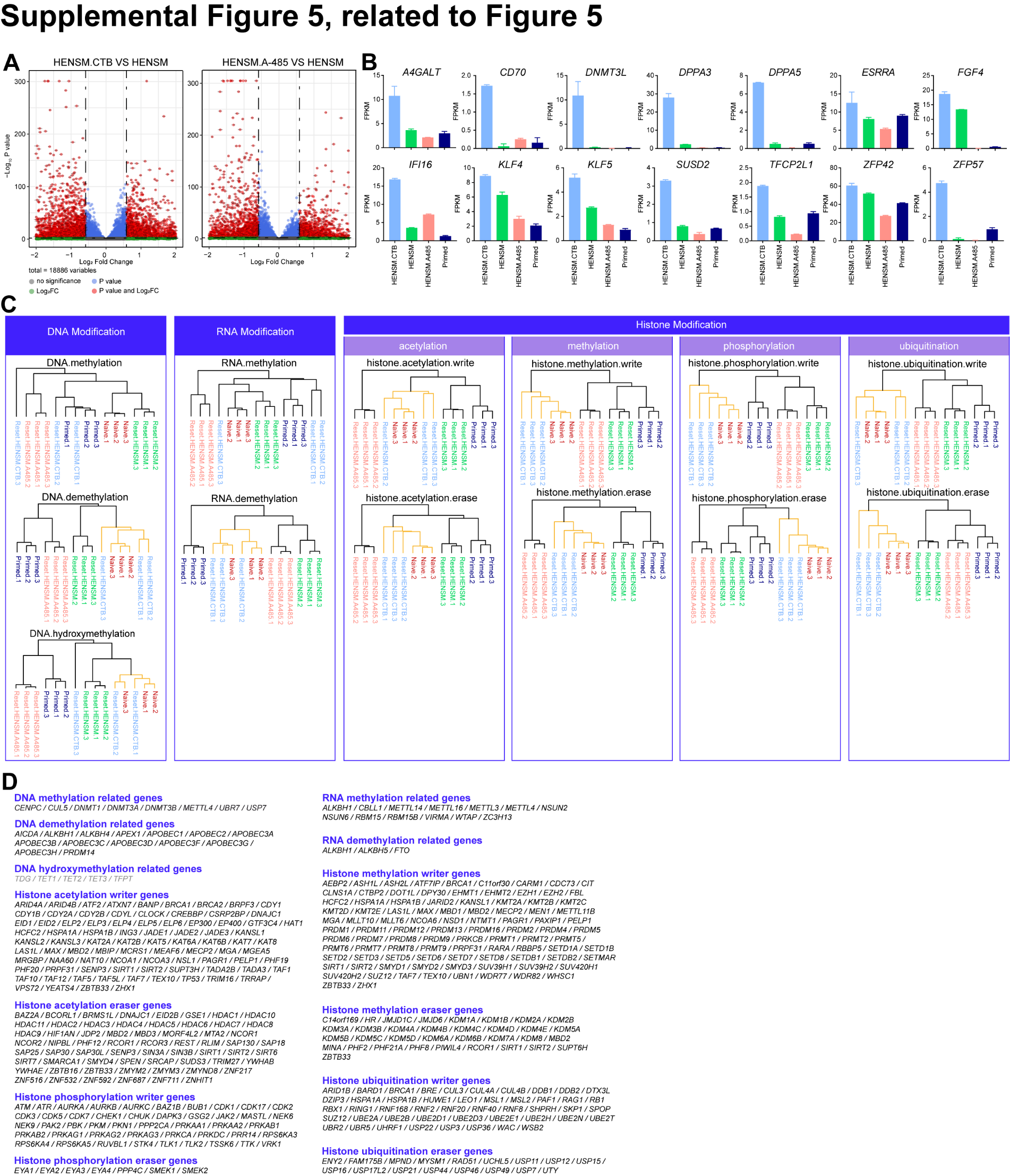
**

*Figure legend on the next page*

**Figure S5**

Differential gene expression patterns induced by H3K27ac activation or inhibition. [Related to Figure 5].

(**A**) Volcano plots displaying the differentially expressed genes between HENSM + CTB and HENSM (left panel), or HENSM + A-485 and HENSM (right panel) resetting cells. X axis represents the differential expressing genes using Log_2_ (fold change of FPKM) between samples. Y axis represents the significance between samples using -Log_10_ P value. (**B**) Histograms showing the differential expression levels of naïve pluripotent genes among HENSM resetting cells with or without A-485 or CTB treatment, compared to primed hiPSCs. (**C-D**) Hierarchical clustering showing the differential expression patterns of multiple epigenetic regulation genes (C), including genes regulating DNA and RNA methylation, histone acetylation, methylation, phosphorylation, and ubiquitination (D).

**
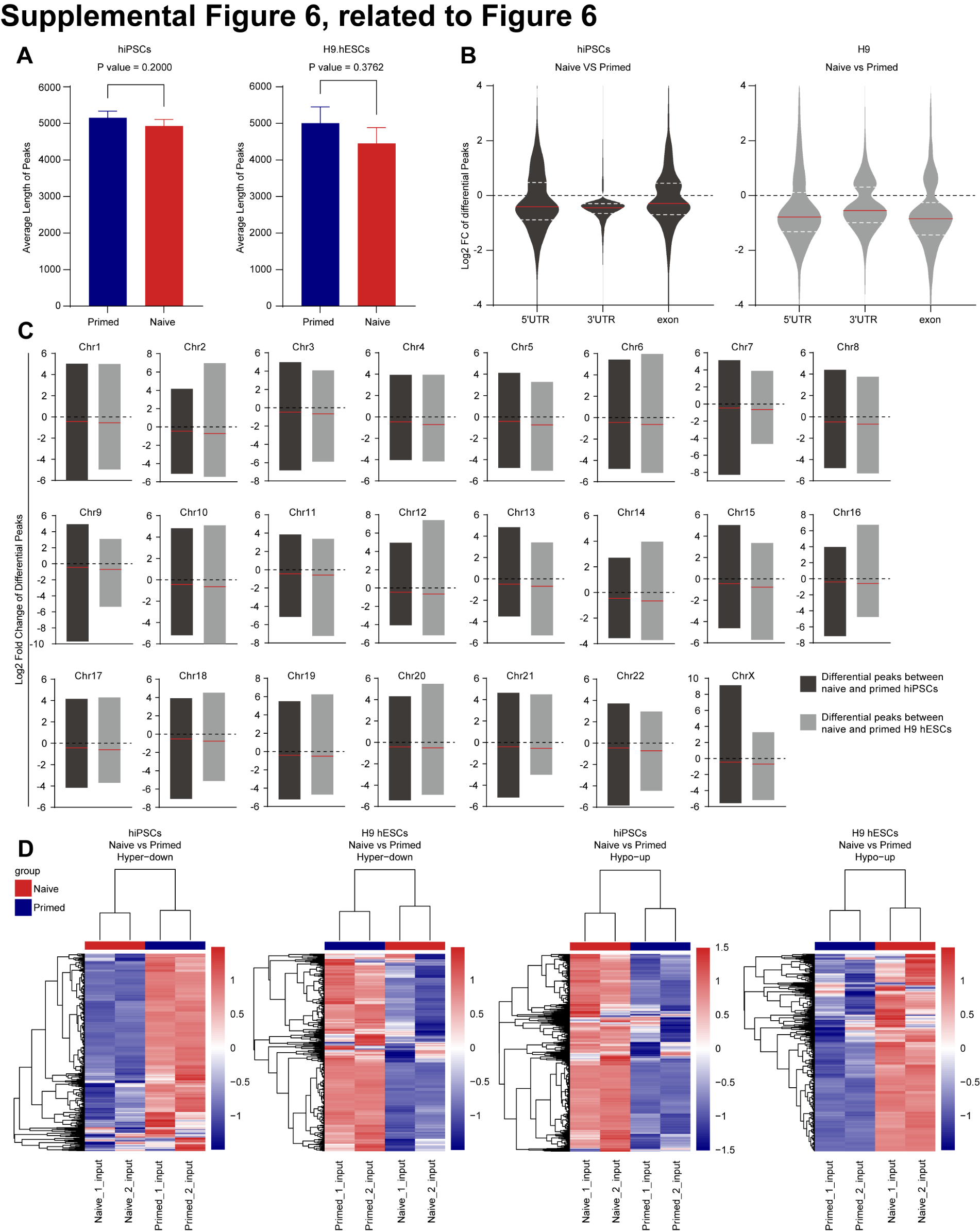
**

*Figure legend on the next page*

**Figure S6**

m6A modification regulates histone acetylation in different pluripotent stem cells. [Related to Fig. 6].

(**A**) Histograms showing the average length of m6A peaks in naive and primed hiPSCs. (**B-C**) Differential m6A peaks in 5’UTR, CDS, and 3'UTR (B) or chromosomes (C) between naive and primed hiPSCs. (**D**) Heatmaps showing the different expression patterns of categorized genes based on the m6A modification.


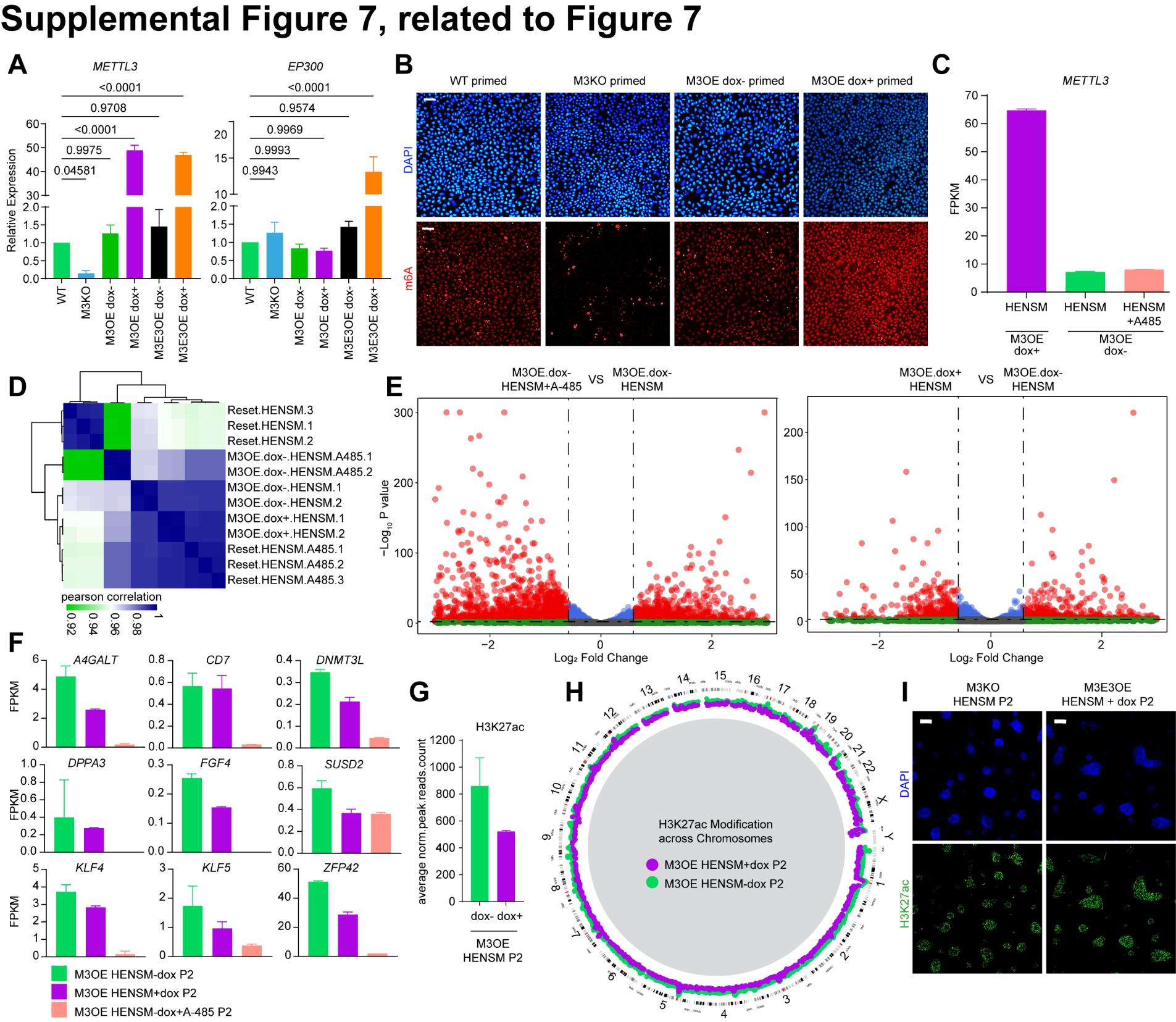


*Figure legend on the next page*

**Figure S7**

METTL3 influences EP300-mediated H3K27ac regulation in naive pluripotency. [Related to Fig. 7].

(**A**) Real-time quantitative gene expression analysis for *METTL3* and *EP300* in WT, M3KO, M3OE (with or without dox treatment), and M3E3OE (with or without dox treatment) primed hiPSCs. Error bars indicate ± 1 SD of technical replicates. (**B**) Representative images of WT, M3KO, and M3OE (with or without dox treatment) primed hiPSCs, detecting m6A levels by IF staining. Scale bars indicate 100 µm. (**C**) Histogram showing the expression levels of *METTL3* in M3OE HENSM resetting cells (dox-, dox+, and dox-A-485+) using FPKM. Error bars indicate ± 1 SD of technical replicates. (**D**) (B) Pearson correlation analysis of samples in Figure S7C. (**E**) Volcano plots displaying the differentially expressed genes between samples. X axis represents the differential expressing genes using Log_2_ (fold change of FPKM) between samples. Y axis represents the significance between samples using -Log_10_ P value. (**F**) Histogram showing the expression levels of naive pluripotent genes in M3OE HENSM resetting cells (dox-, dox+, and dox-A-485+) using FPKM. Error bars indicate ± 1 SD of technical replicates. (**G**) Histogram displaying the average normalized H3K27ac peak counts between M3OE HENSM resetting cells with or without dox treatment. Error bars indicate ± 1 SD of technical replicates. (**H**) H3K27ac peaks across the chromosomes between samples. Each dot represents a peak. (**I**) Representative images of M3KO and M3E3OE + dox HENSM resetting cells, detecting H3K27ac levels by IF staining. Scale bars indicate 100 µm.
